# Unraveling the interaction between the phageome and bacteriome in the rumen and its role in influencing metabolome dynamics in dairy cows at different lactation stages

**DOI:** 10.1186/s40168-025-02260-1

**Published:** 2025-12-15

**Authors:** Mengya Wang, Chenguang Zhang, Lichao Zhao, Qingyan Yin, Zhijie Cui, Xiaodong Chen, Jianrong Ren, Yue Wang, Ming Xu, Yangchun Cao, Shengru Wu, Junhu Yao

**Affiliations:** 1 College of Animal Science and Technology, Northwest A&F University, Yangling, Shaanxi 712100 People’s Republic of China; 2 Faculty of Land and Food Systems, the University of British Columbia, Vancouver, BC V6T 1Z4 Canada; 3 College of Animal Science, Inner Mongolia Agricultural University, Hohhot, 010018 People’s Republic of China

**Keywords:** Rumen phages, Dynamics, Rumen microbiome, Metabolome, Milk protein, Milk yield, Dairy cows

## Abstract

**Background:**

Although the roles of rumen microbiome in milk yield and milk protein synthesis have been widely recognized, knowledge on how ruminal microbiome dynamic changes affect these two traits during the whole lactation is lacking. Phages have been shown to affect the microbiota, but little is known about the shift patterns of ruminal phages and if they may modulate rumen microbiome during lactation. Herein, a longitudinal study was performed to identify the potential roles of ruminal phageome and bacteriome interactions, and metabolic function shift in affecting milk yield and protein content using metagenomic and metabolomic profiling of rumen microbiome from the peak, early, and later mid-lactation stages.

**Results:**

A total of 780 ruminal bacterial phages were identified, which exhibited two primary shifting patterns: (1) decreasing then increasing; (2) decreasing then stabilizing through the lactation. Bacteriome also showed first increasing then stabilizing or continuously declining besides exhibiting two similar shifting patterns to those of phages. By associating the differentially abundant phages with their host bacteria, we observed that significantly increased *Lactococcus phage BM13*, *Corynebacterium phage P1201*, and *Campylobacter phage CJIE4-5* in peak lactation, along with *Lactobacillus phage Lv-1* in early and later mid-lactation, were positively correlated with the relative abundance of their hosts. However, significantly increased *Bacillus phage BCU4* and the *Enterococcus phage phiNASRA1* in early mid-lactation were negatively related to their host abundance. In terms of bacteria, *Ruminococcus flavefaciens* and *Faecalibacterium* sp. *CAG 74* had the highest abundance in peak lactation, whereas most *Prevotella* species were more abundant in early and later mid-lactation. Notably, ruminal carbohydrate and amino acid metabolism functions were enhanced in early mid-lactation. Further structural equation model and network analysis revealed that abundant *Bacillus phage BCU4* and *Enterococcus phage phiNASRA1* in early mid-lactation were associated with increased relative abundance of *Prevotella* species, possibly due to a reduction in *Bacillus cereus* and *Enterococcus faecalis*. Additionally, these *Prevotella* species exhibited positive relationships with rumen metabolites, such as L-phenylalanine, phenylacetylglycine, N-acetyl-D-phenylalanine, and propionate content, which contributed to the improved milk protein yield.

**Conclusions:**

This study revealed the bacteriome and phageome interactions at different lactation stages, and the key phages and bacteria regulating the rumen function and metabolism thus contributing to the milk traits of cows. The potential regulatory roles of phages in affecting the rumen bacteriome suggest that they can be powerful targets for future interventions to improve rumen functions.

Video Abstract

**Supplementary Information:**

The online version contains supplementary material available at 10.1186/s40168-025-02260-1.

## Introduction

The rumen microbiome and its contribution to variations in milk phenotypes, including milk yield and milk protein synthesis at the compositional and functional levels, have recently been reported [1, 2]. However, although previous studies mainly focused on the mid-lactation, it is unclear whether such contribution can be persistent through the entire lactation period of dairy cows. Although some efforts have been made to assess the temporal shift in the rumen microbiota, such as transition period (the 3 weeks before and the 3 weeks after calving), cows need to adapt to changes from non-lactating to lactating and diet alterations (high forage to high concentrate) during this period [3, 4]. It is therefore difficult to definitively discern the effects of natural physiological state versus dietary interventions on the rumen microbiota. Moreover, instability of bacterial and archaeal communities in the rumen of dairy cows during lactation has been reported, despite the same feeding and management conditions [5–7]. These studies have demonstrated that certain rumen microbial taxa are related to feed efficiency, methane emissions, and milk performance, suggesting that rumen microbes play a vital role at different stages of lactation in dairy cows. However, the changes in other rumen microbial groups such as eukaryotes and viruses, especially phages during lactation, as well as the temporal dynamics of microbial function on milk performance are not well understood.

Recent studies have identified that phageomes (usually defined as all bacterial phages within a particular microbiome) in the rumen are among the most abundant entities in that microecosystem [8, 9]. Phages have the ability to affect the abundance of target bacterial hosts because of their generally host specificity at the species level [10–14]. In addition, phages can also lead to alterations of non-susceptible bacteria through competition of inter-bacteria or niches, thereby driving the microbial richness and diversity [15, 16]. There are also studies indicating that phages play crucial roles in shaping microbial community composition and regulating host bacterial metabolism [17–19]. Although the role of phages in rumen regulation of microbiome has been reported in different diets and animal breeds [8, 20, 21], little is known about rumen phageome shift in dairy cows during the lactation stages and how they may affect rumen fermentation by influencing their bacterial hosts.

We speculated that the phageome has dissimilar shift patterns to those of the bacteriome and that phageome dynamics can affect bacteria to result in the metabolome changes, leading to varied contributions to milk phenotypes. Therefore, in this study, we aimed to reveal the potential role of phageome in dynamic alterations in the rumen microbiome and metabolome during lactation of dairy cows using a longitudinal multiomics comparison of metagenomic and metabolomic profiles of Holstein cows with similar body conditions at 70 (peak lactation), 120 (early mid-lactation), and 150 (later mid-lactation) days after calving. Meanwhile, a structural equation model (SEM) was used to analyze the interactions between rumen phages and bacteria, as well as the underlying mechanisms through which the rumen metagenome influences the metabolome and milk protein yield. The present study provides a fundamental understanding of bacteriome-phageome within the rumen for future rumen microbiome intervention strategies.

## Results

### Ruminal phageome and bacteriome profiles and diversity during different lactation stages

**Fig. 1:**
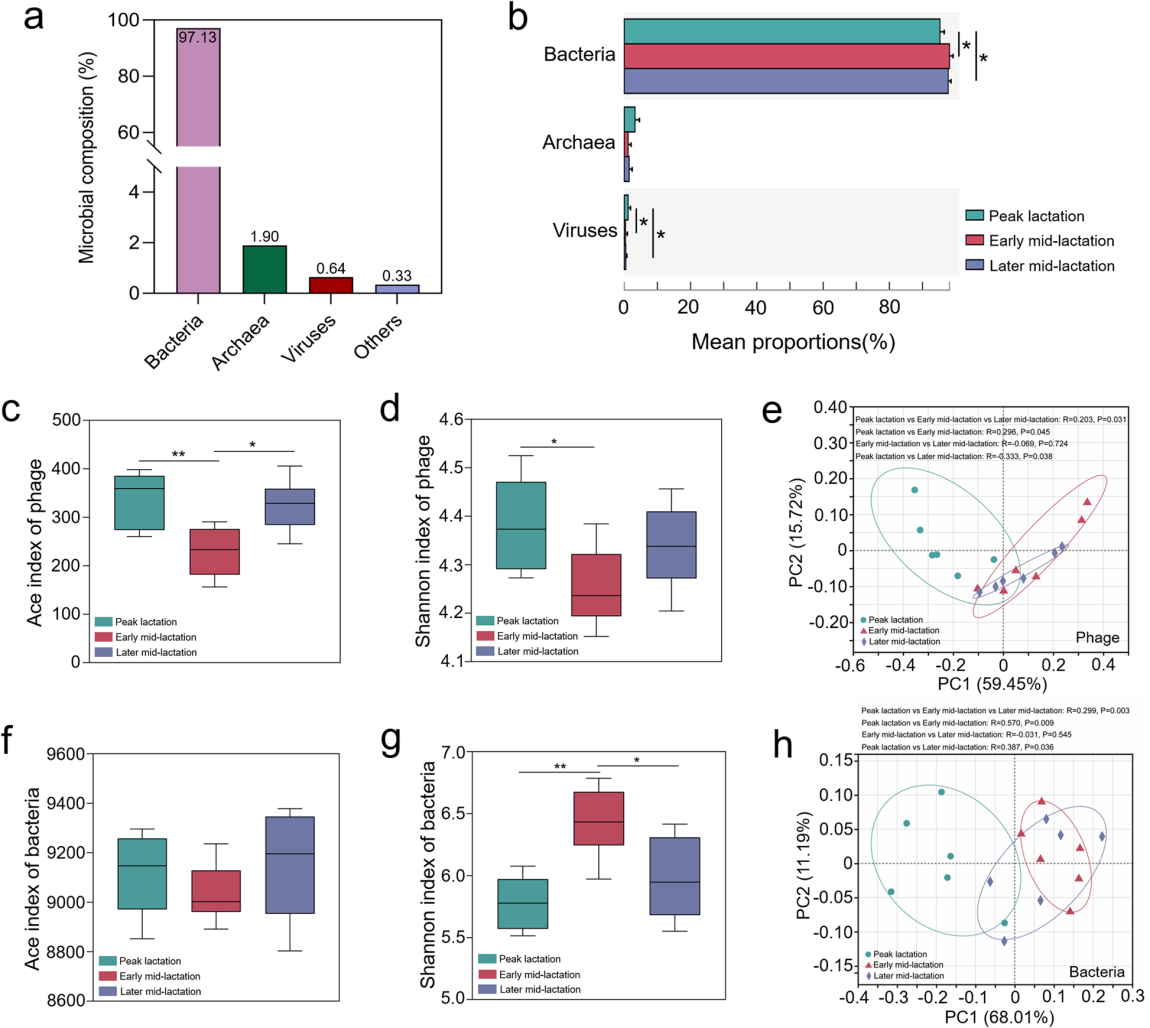
Comparison of rumen microbial composition profiles and diversity from dairy cows at different lactation stages. **a** Profiles of the rumen microbial composition. **b** Comparison of rumen microbial domain levels. Ace (**c**) and Shannon (**d**) indices of rumen phages. **e** Rumen phage signatures based on species visualized using principal coordinate analysis (PCoA). Ace (**f**) and Shannon (**g**) indices of rumen bacteria at species level. **h** Rumen bacterial signatures based on species visualized via PCoA. *P* values were determined using the nonparametric Kruskal–Wallis test. *P < 0.05, **P < 0.01

Metagenome sequencing generated a total of 1,198,046,164 reads. After quality control, removing host genes, and de novo assembly, a total of 14,598,101 contigs were obtained (the N50 length of 739 ± 15 bp), with 811,006 ± 26,126 per sample (Table S1). Metagenomic profiling of dairy cows at three lactation stages revealed that rumen microbiome consisted of 97.13% bacteria, 1.90% archaea, 0.64% viruses (mainly phages), and 0.33% other microbes (Fig. 1a). Specifically, the relative abundance of bacteria was significantly increased, while phages was significantly decreased in early and later mid-lactation compared to peak lactation (*P* < 0.05, Fig. 1b). No difference in the relative abundance of archaea was observed among the three lactation stages (*P* > 0.05, Fig. 1b). At the species level, the microbial alpha diversity results suggested that Ace index of phages (*P* < 0.01, Fig. 1c) and archaea (*P* < 0.05, Fig. S1b), as well as Shannon index of phages (*P* < 0.05, Fig. 1d) in early mid-lactation, were significantly lower than those of peak lactation, whereas the Shannon index of bacteria in the early mid-lactation was significantly higher (*P* < 0.01, Fig. 1g). Bacterial Ace index (Fig. 1f) and archaeal Shannon index (Fig. S1c) were not different among the various stages of lactation.

Principal coordinate analysis (PCoA) plot exhibited spatial separations for beta-diversity of ruminal phages (*P* < 0.05, Fig. 1e) and bacteria (*P* < 0.01, Fig. 1h) during the three lactation stages, while no separation was observed in archaea species (Fig. S1d). In the pairwise comparisons of these three stages, there were also obvious dissimilarity in the beta-diversity of phages (Fig. 1e) and bacteria (Fig. 1h) (peak lactation vs. early/later mid-lactation, *P* < 0.05).

### Dynamics of rumen phageome and bacteriome in dairy cows during the lactation stages

**Fig. 2:**
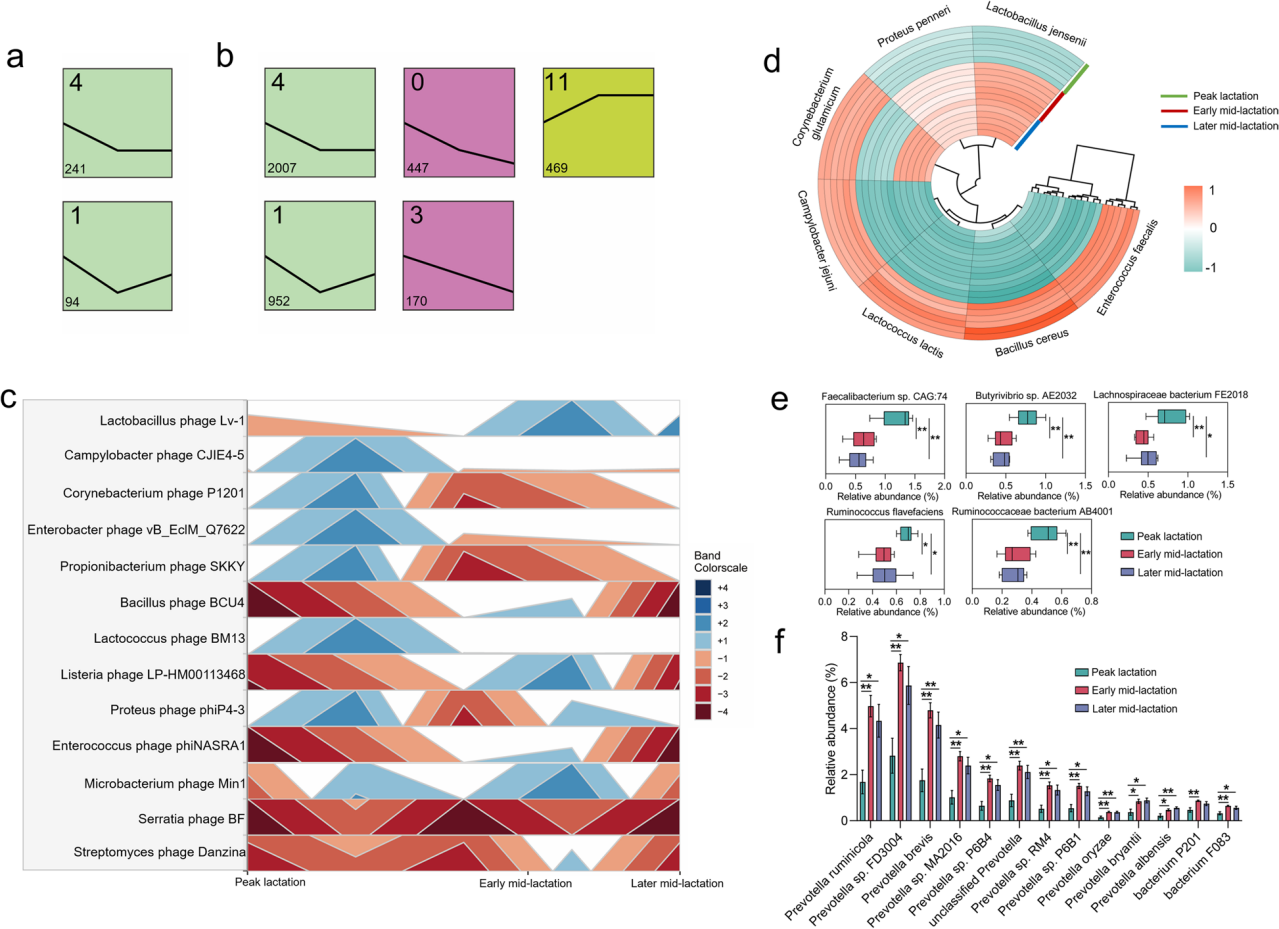
The shift patterns of rumen phages and bacteria in cows at different lactation stages. **a** The dominant shift patterns of rumen phages as changes of the lactation stage. **b** The main shift patterns of rumen bacteria as changes of the lactation stage. In each frame, the profile ID is presented on the top left, the number of phages or bacteria is shown on the bottom left, the *x*-axis indicates the stages of lactation, and the *y*-axis denotes the abundance of phages or bacteria. **c** The dynamic alterations of differential phages at different lactation stages. Bands: The *x*-axis is a time series, and the *y*-axis is the relative abundance of a phage at a given timepoint, collapsed to a more uniform height. Colors represent quartiles relative to the median. **d** The heatmap shows the differences in host bacteria corresponding to different phages during the three periods. Differentially abundant bacterial species in peak lactation (**e**), early, and later mid-lactation (**f**). *P < 0.05, **P < 0.01

A total of 781 ruminal phages were identified among the three different lactation stages, of which 780 were bacterial phages, and only one was archaeal phage (i.e., *Halobacterium phage ChaoS9*). Short Time-series Expression Miner (STEM) analysis based on phageome revealed cluster of 16 shift patterns (profile 0–15, Fig. S2a–b, the meaning of Fig. S2b: shift patterns of the identified phages at different lactation stages, and the colored frame represents the number of allocated phages with statistical significance) from peak lactation to later mid-lactation, with profile 4 (decreasing then stabilizing) and profile 1 (decreasing then increasing) being the primary shift patterns (higher number of phages assigned, *P* < 0.05), containing 241 and 94 phages, respectively (Fig. 2a). As the phageome changed, the bacteriome underwent 16 different shift patterns (profile 0–15, Fig. S2c–d, the meaning of Fig S2d is similar to the meaning of phages mentioned above), of which 5 patterns were considered as significant (as defined above for the primary shift patterns of phageome, *P* < 0.05) (Fig. 2b). Specifically, the most abundant profile 4 (decreasing then stabilizing) and profile 1 (decreasing then increasing) containing 2007 and 952 bacteria were consistent with the transfer patterns of phageome (profile 4 and 1). In contrast, profile 11 containing 469 bacteria showed an initial increase and subsequently tended to stabilize. Additionally, the bacteriome continuously declined with the progression of lactation stage (i.e., profiles 0 and 3 containing 447 and 170 bacteria) (Fig. 2b).

For the comparison of rumen differential phages, the relative abundances of *Corynebacterium phage P1201*, *Propionibacterium phage SKKY*, and *Proteus phage phiP4-3* showed decrease then increase from peak to later mid-lactation (peak lactation vs. early/later mid-lactation, early mid-lactation vs. later mid-lactation, *P* < 0.05, Fig. 2c). On the contrary, the relative abundances of *Bacillus phage BCU4* and *Enterococcus phage phiNASRA1* (peak lactation vs. early/later mid-lactation, early mid-lactation vs. later mid-lactation, *P* < 0.05), as well as *Listeria phage LP-HM00113468*, *Microbacterium phage Min1*, and *Streptomyces phage Danzina* (peak lactation vs. early mid-lactation, early mid-lactation vs. later mid-lactation, *P* < 0.05; peak lactation vs. later mid-lactation, *P* > 0.05), exhibited increase then decrease during the lactation (Fig. 2c). Interestingly, compared with peak lactation, the relative abundance of *Lactobacillus phage Lv-1* significantly increased in both early and later mid-lactation, while the abundances of *Campylobacter phage CJIE4-5*, *Enterobacter phage vB_EclM_Q7622*, and *Lactococcus phage BM13* significantly reduced in both early and later mid-lactation (*P* < 0.05, Fig. 2c). Moreover, the differential hosts of these phages were further evaluated and revealed that the relative abundances of *Bacillus cereus* and *Enterococcus faecalis* were decreased then increased from peak to later mid-lactation (peak lactation vs. early/later mid-lactation, early mid-lactation vs. later mid-lactation, *P* < 0.05, Fig. 2d). *Campylobacter jejuni*, *Lactococcus lactis*, and *Corynebacterium glutamicum* in peak lactation had an obvious increased relative abundance than in early and later-mid lactation (*P* < 0.05), while the relative abundances of *Lactobacillus jensenii* and *Proteus penner* in early and later-mid lactation (*P* < 0.05) significantly increased compared to peak lactation (Fig. 2d).

For the comparison of rumen bacteria, the relative abundances of *Faecalibacterium* sp. *CAG 74*, *Butyrivibrio* sp. *AE2032*, *Lachnospiraceae bacterium FE2018*, *Ruminococcus flavefaciens*, and *Ruminococcaceae bacterium AB4001* were significantly increased in peak lactation than in early and later mid-lactation (*P* < 0.05, Fig. 2e). Compared with peak lactation, the relative abundances of 11 *Prevotella* species (such as *Prevotella ruminicola*, *Prevotella* sp. *FD3004*, *Prevotella brevis*, *Prevotella* sp. *MA2016*, and *Prevotella* sp. *P6B4*), *bacterium F083*, and *bacterium P201* (Fig. 2f) were significantly increased in early and/or later mid-lactation (*P* < 0.05). For the relative abundances of archaea, *Methanobrevibacter YE315* and *Methanobrevibacter smithii* were significantly reduced in early and later mid-lactation (*P* < 0.05, Fig. S2e-f).

### Rumen microbial functions during the different lactation stages

**Fig. 3:**
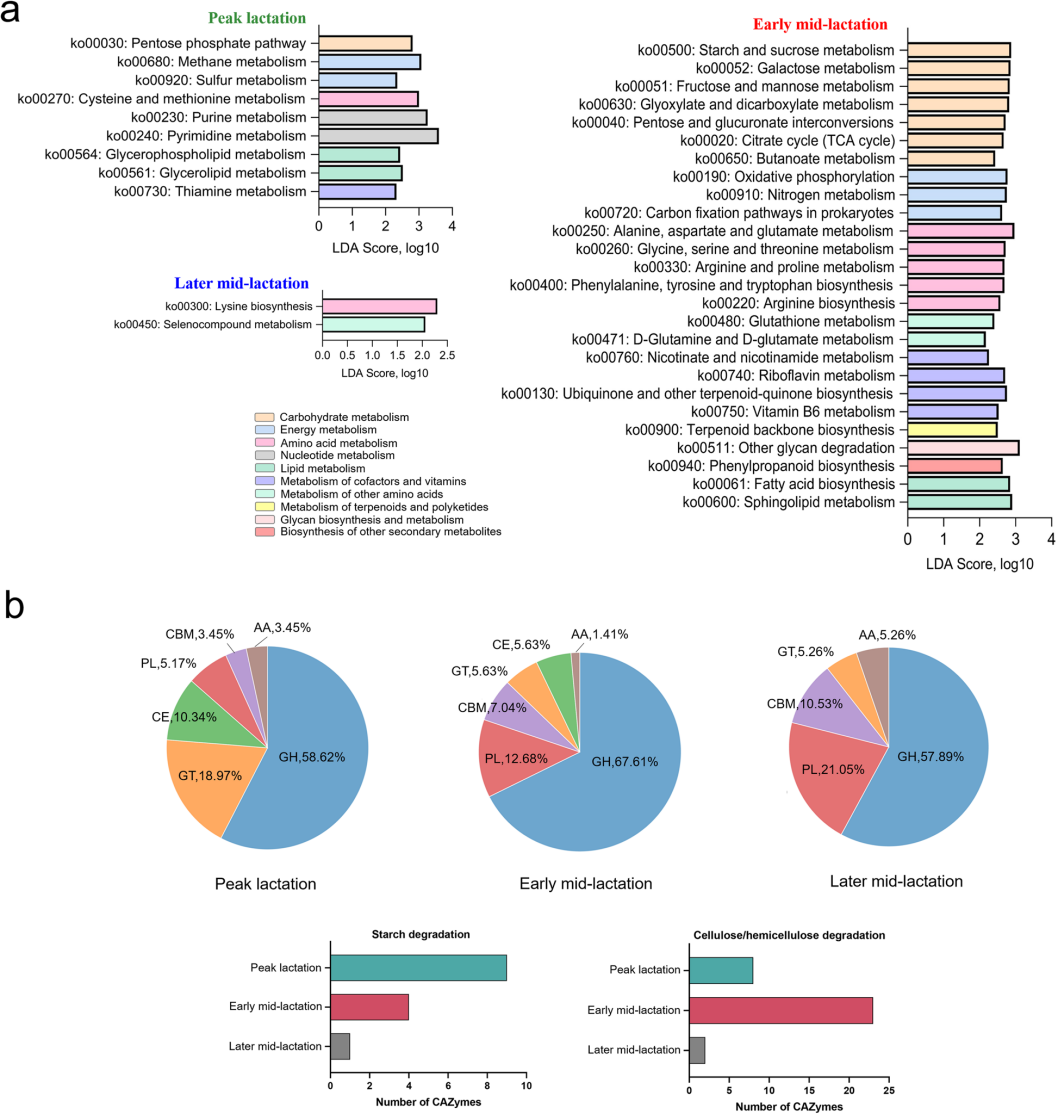
Differential KEGG and CAZyme functions of dairy cows at different lactation stages. **a** Significantly enriched microbial metabolic pathways. Significant differences were tested by linear discriminant analysis effect size (LEfSe) analysis, with a linear discriminant analysis (LDA) score > 2 and a *P* value < 0.05. **b** Proportion and numbers of genes encoding CAZymes. GH, glycoside hydrolase; GT, glycosyltransferase; PL, polysaccharide lyase; CE, carbohydrate esterase; CBM, carbohydrate-binding module; AA, auxiliary activity

The overall functional PCoA of Kyoto Encyclopedia of Genes and Genomes (KEGG) showed significant differences among the three lactation stages (ANOSIM: *P* < 0.01), and the pairwise comparison of the three periods exhibited clear separations (peak lactation vs. early/later mid-lactation, ANOSIM *P* < 0.05, Fig. S3a). In total, 37 differential metabolic pathways were identified, of which 26 were enriched in early mid-lactation (LDA > 2, *P* < 0.05, Fig. 3a), including seven “carbohydrate metabolism” pathways (such as starch, sucrose metabolism, fructose, mannose metabolism, and galactose metabolism), seven “amino acid metabolism” pathways (such as phenylalanine, tyrosine, tryptophan biosynthesis, alanine, aspartate, glutamate metabolism, and arginine biosynthesis), four “cofactor and vitamin metabolism” pathways (vitamin B6 metabolism, riboflavin metabolism, nicotinate, nicotinamide metabolism, ubiquinone, and other terpenoid-quinone biosynthesis), three “energy metabolism” pathways (nitrogen metabolism, oxidative phosphorylation, and carbon fixation pathways in prokaryotes), two “lipid metabolism” pathways (fatty acid biosynthesis, and sphingolipid metabolism), one “terpenoids and polyketides metabolism” pathway (terpenoid backbone biosynthesis), one “glycan biosynthesis and metabolism” pathway (other glycan degradation), and one “other secondary metabolite biosynthesis” pathway (phenylpropanoid biosynthesis). In peak lactation, 9 pathways were significantly enriched (LDA > 2, *P* < 0.05), including two “energy metabolism” pathways (methane metabolism and sulfur metabolism), two “nucleotide metabolism” pathways (purine metabolism and pyrimidine metabolism), two “lipid metabolism” pathways (glycerolipid metabolism and glycerophospholipid metabolism), one “carbohydrate metabolism” pathway (pentose phosphate pathway), one “amino acid metabolism” pathway (cysteine and methionine metabolism), and one “cofactor and vitamin metabolism” pathway (thiamine metabolism). Notably, only two pathways (lysine biosynthesis and selenocompound metabolism) related to amino acid metabolism were significantly enriched in later mid-lactation (LDA > 2, *P* < 0.05, Fig. 3a).

The overall functional PCoA of carbohydrate-active enzyme (CAZyme) indicated obvious separations among the three lactation stages (ANOSIM *P* < 0.01), and the pairwise comparison of the three periods showed conspicuous separations (peak lactation vs. early/later mid-lactation, ANOSIM *P* < 0.05, Fig. S3b). A total of 148 differential genes encoding CAZymes were identified during the three lactation stages (LDA > 2, *P* < 0.05, Fig. 3b, Fig. S4). Among them, 71 CAZyme genes were enriched in early mid-lactation, including 48 glycoside hydrolases (GHs, 67.61%), 9 polysaccharide lyases (PLs, 12.68%), 5 carbohydrate-binding modules (CBMs, 7.04%), 4 glycosyltransferase (GTs, 5.63%), 4 carbohydrate esterases (CEs, 5.63%), and 1 auxiliary activity (AA, 1.41%). 58 CAZyme genes were enriched in peak lactation, including 34 GHs (58.62%), 11 GTs (18.97%), 6 CEs (10.34%), 3 PLs (5.17%), 2 CBMs (3.45%), and 2 AAs (3.45%). Nineteen CAZyme genes were enriched in later mid-lactation, including 11 GHs (57.89%), 4 PLs (21.05%), 2 CBMs (10.53%), 1 GT (5.26%), and 1 AA (5.26%). Since GHs accounted for the highest proportion in the rumen at different periods, further classification of GH-coding genes showed that GH13 families, GH77, and GH31 involved in starch degradation were significantly enriched in peak lactation and early mid-lactation, especially in peak lactation (LDA > 2, *P* < 0.05; Fig. 3b, Fig. S4). Meanwhile, GH5, GH8, GH10, GH30, and GH43 families, which are responsible for cellulose/hemicellulose degradation, were significantly enriched in peak lactation and early mid-lactation, especially in early mid-lactation. In comparison, the gene enzymes related to starch degradation (GH13_7) and cellulose/hemicellulose degradation (GH30_2, GH43_3) were relatively less abundant in later mid-lactation.

### Rumen microbial metabolome at different lactation stages

**Fig. 4:**
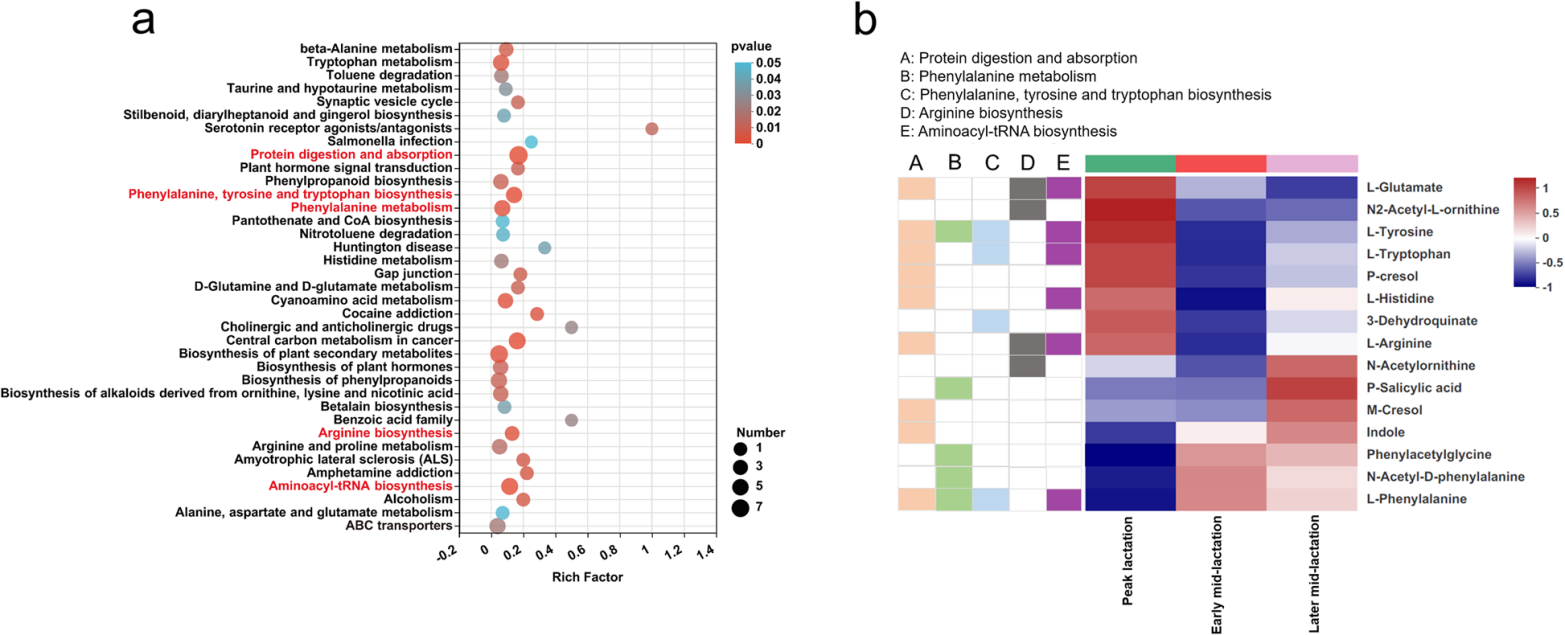
Changes in the rumen metabolomic profiles of dairy cows at different lactation stages. **a** KEGG pathway enrichment analysis of differential rumen metabolites. **b** Alterations of differential metabolites among the three periods that were involved in protein digestion and absorption; phenylalanine, tyrosine and tryptophan biosynthesis; phenylalanine metabolism; arginine biosynthesis; and aminoacyl-tRNA biosynthesis

A total of 1120 compounds were annotated in the rumen metabolome. Through the partial least squares discriminant analysis (PLS-DA), the metabolome profiles of three lactation stages were separated well (Fig. S5a). Rumen metabolic pathway analysis based on differential metabolites revealed the enrichment of 37 pathways, in which “protein digestion and absorption,” “phenylalanine, tyrosine and tryptophan biosynthesis,” “phenylalanine metabolism,” “arginine biosynthesis,” and “aminoacyl-tRNA biosynthesis” were the most significantly different pathways (*P* < 0.01, Fig. 4a, Fig. S5b). We further compared the differential metabolites involved in these significantly enrichment pathways (Fig. 4b), and the results showed that compared with peak lactation, the relative abundances of phenylacetylglycine and L-phenylalanine were significantly increased in early and later mid-lactation, while L-glutamate, N2-acetyl-l-ornithine, L-tyrosine, L-tryptophan, and P-cresol were significantly reduced (*P* < 0.05). N-acetyl-d-phenylalanine had a higher relative abundance in early mid-lactation than in peak lactation, but L-histidine, 3-dehydroquinate, and L-arginine exhibited the opposite abundance trend (*P* < 0.05). The relative abundance of P-salicylic acid was significantly increased in later mid-lactation compared to peak lactation and early mid-lactation (*P* < 0.05). In addition, the ruminal fermentation parameters of the cows from different lactation stages were evaluated (Table S2). The acetate proportion was significantly increased in peak lactation compared to early and later mid-lactation (*P* < 0.05). Meanwhile, the propionate proportion was significantly higher in early mid-lactation compared with later mid-lactation (*P* < 0.05).

### Identifying key bacteria contributing to milk traits

**Fig. 5:**
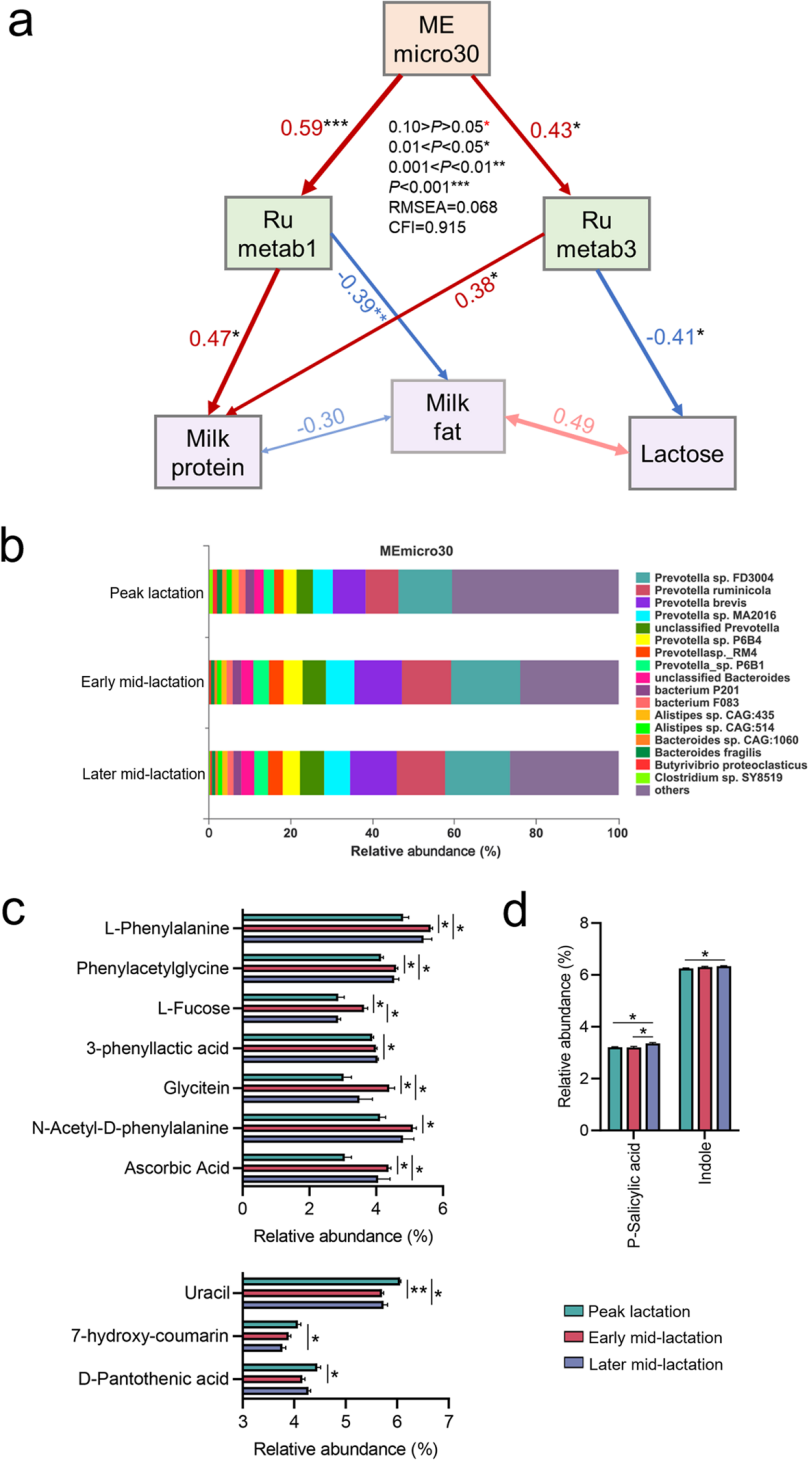
Effective pathways through which the ruminal microbiome affects milk composition. **a** Structural equation model (SEM) was established by the milk composition and modules of the ruminal microbiome (at the species level) and the ruminal metabolome in the WGCNA. The numbers adjacent to the arrows indicate the effective size of the relationship. The red and blue arrows represent positive and negative paths, respectively. RMSEA, root mean square error of approximation; CFI, comparative fit index. **b** Microbial composition of the rumen metagenome module 30 (MEmicro30). **c** Metabolites of differences in rumetab 1. **d** Metabolites of differences in rumetab 3. *P < 0.05, **P < 0.01, ***P < 0.001

Compared with later mid-lactation, the milk yield was significantly increased in peak lactation and early mid-lactation (*P* < 0.05). Meanwhile, the milk protein content was significantly increased in both the early (*P* < 0.05) and later mid-lactation (*P* < 0.01) than in peak lactation (Fig. S6). Structural equation modeling (SEM, see the “Methods” section) based on weighted gene co-expression network analysis (WGCNA) was established to explore the relationship between different omics and milk components (Fig. 5a, Fig. S7a–b). The results suggested that ruminal metagenome module 30 (MEmicro30), composed mainly of *Prevotella* species, had a positive correlation with ruminal metabolome modules 1 (rumetab 1) and 3 (rumetab 3) (Fig. 5a–b). Moreover, these two metabolic modules were positively associated with milk protein and negatively correlated with milk fat and lactose, respectively (*P* < 0.05). Further analysis of the metabolites involved in the significantly different pathways of rumetab 1 revealed that the relative abundances of L-phenylalanine, phenylacetylglycine, and ascorbic acid were significantly increased in early and later mid-lactation compared to those in peak lactation, whereas uracil was significantly reduced (*P* < 0.05, Fig. 5c, Table S3, Fig. S8a). The relative abundances of P-salicylic acid and indole participated in the differential metabolic pathways of rumetab 3 were significantly increased in later mid-lactation than in peak lactation (*P* < 0.05, Fig. 5d, Table S4, Fig. S8b).

**Fig. 6:**
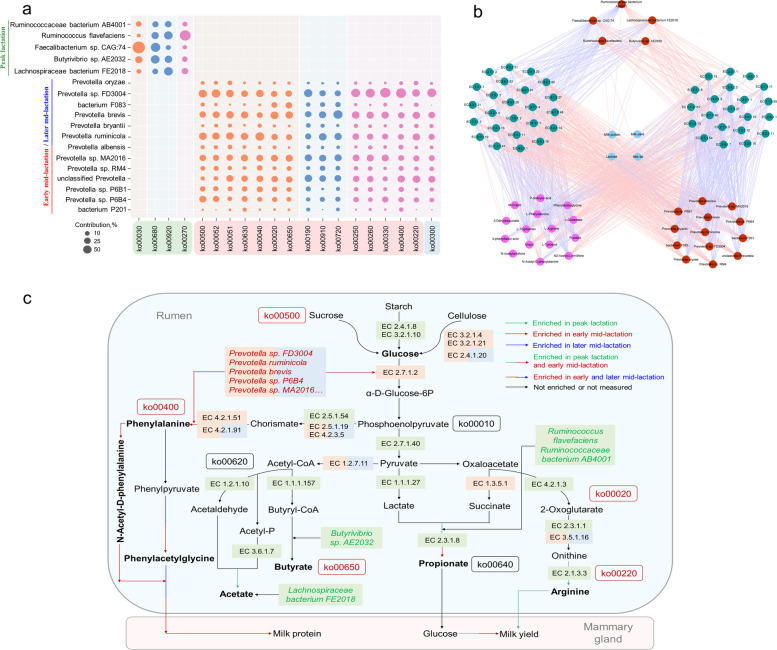
Multiomics integration included the rumen microbiome, microbial functions, rumen metabolome, and milk composition. **a** Contribution of differential ruminal bacteria to the identified KEGG pathways. Colors of the circle: orange, carbohydrate degradation; blue, energy metabolism; and purple, amino acid biosynthesis. The size of the circle is positively related to its contribution. Colors of microbial metabolic pathway (ko number): green, enriched in peak lactation; red, enriched in early mid-lactation; blue, enriched in later mid-lactation. ko00030, pentose phosphate pathway; ko00680, methane metabolism; ko00920, sulfur metabolism; ko00270, cysteine and methionine metabolism; ko00500, starch and sucrose metabolism; ko00052, galactose metabolism; ko00051, fructose and mannose metabolism; ko00630, glyoxylate and dicarboxylate metabolism; ko00040, pentose and glucuronate interconversions; ko00020, citrate cycle; ko00650, butanoate metabolism; ko00190, oxidative phosphorylation; ko00910, nitrogen metabolism; ko00720, carbon fixation pathways in prokaryotes; ko00250, alanine, aspartate and glutamate metabolism; ko00260, glycine, serine and threonine metabolism; ko00330, arginine and proline metabolism; ko00400, phenylalanine, tyrosine and tryptophan biosynthesis; ko00220, arginine biosynthesis; ko00300, lysine biosynthesis. **b** Spearman correlation network of the rumen bacterial species, genes encoding enzymes, rumen metabolites, and milk compositions. The colors of nodes are as follows: red, rumen bacteria; green, genes encoding enzymes; purple, rumen metabolites; and blue, milk composition. Line colors: red, positive; blue, negative. Only strong correlations are displayed (|R|> 0.5, *P* < 0.05). **c** Microbial functions and species involved in the key carbohydrate metabolism and amino acid metabolism in dairy cows at different lactation stages. Comparisons of the relative abundance of genes encoding enzymes related to key metabolic pathways for carbohydrate fermentation and amino acid biosynthesis were performed using the Kruskal–Wallis test

Ruminants require effective carbohydrate dissociation to meet their energy needs. Amino acids provide substrates for milk protein synthesis. We further focused on the contribution of differential bacteria or SEM-selected bacteria to the identified carbohydrate, energy, and amino acid metabolism pathways. *Faecalibacterium* sp. *CAG:74*, which significantly increased in peak lactation, had a higher contribution (comparing the contribution values of different bacteria to the same metabolic pathway, the contribution values were relatively high and showed significant differences from that of other bacteria to this pathway, *P* < 0.05) to pentose phosphate pathway (ko00030, contribution value, CoVa, 65.83%), and *Ruminococcus flavefaciens* had greater contributions to sulfur metabolism (ko00920, CoVa, 24.09%) cysteine and methionine metabolism (ko00270, CoVa, 34.52%) pathways (Fig. 6a). Nevertheless, *Prevotella* species that increased significantly in early and later mid-lactation, such as *Prevotella* sp. *FD3004*, *Prevotella brevis*, and *Prevotella ruminicola*, had higher contributions to carbohydrate, energy, and amino acid metabolism pathways simultaneously, including starch and sucrose metabolism (ko00500), galactose metabolism (ko00052), nitrogen metabolism (ko00910), arginine, proline metabolism (ko00330), and phenylalanine, tyrosine, tryptophan biosynthesis (ko00400) pathways (10.72% < CoVa < 32.03%).

Spearman’s rank correlations among the differential/SEM-selected bacterial species, gene-coding enzymes involved in key KEGG pathways, differential or SEM-selected metabolites, and milk performance were assessed (Fig. 6b). Positive relationships exhibited between five bacterial species (*Ruminococcaceae bacterium AB4001*, *Lachnospiraceae bacterium FE2018*, *Faecalibacterium* sp. *CAG:74*, *Ruminococcus flavefaciens*, and *Butyrivibrio* sp. *AE2032*) and acylphosphatase (EC3.6.1.7), acetaldehyde dehydrogenase (EC1.2.1.10), ornithine carbamoyltransferase (EC2.1.3.3), and L-lactate dehydrogenase (EC1.1.1.27) (*R* > 0.50, *P* < 0.05). Ten *Prevotella* species (*Prevotella ruminicola*, *Prevotella* sp. *MA2016*, *Prevotella* sp. *FD3004*, *Prevotella brevis*, *Prevotella* sp. *P6B4*, *Prevotella oryzae*, *Prevotella* sp. *RM4*, *Prevotella albensis*, *Prevotella* sp. *P6B1*, and *Prevotella bryantii*) were positively associated with enzymes participating in carbohydrate and amino acid metabolism, such as succinate dehydrogenase (EC1.3.5.1), cellulase (EC3.2.1.4), beta-glucosidase (EC3.2.1.21), arogenate dehydratase (EC4.2.1.91), prephenate dehydratase (EC4.2.1.51), and glucokinase (EC2.7.1.2) (*R* > 0.50, *P* < 0.05). The correlations between metabolites and milk traits revealed positive correlations between L-phenylalanine, phenylacetylglycine, N-acetyl-D-phenylalanine, and milk protein, while L-arginine was positively related to milk yield (*R* > 0.50, *P* < 0.05). In addition, significant associations (|*R*|> 0.50, *P* < 0.05) between these metabolites and gene-coding enzymes (EC3.6.1.7, EC2.1.3.3, EC1.1.1.27, EC1.3.5.1, EC3.2.1.4, EC4.2.1.51, and EC4.2.1.91) were also observed (Fig. 6b–c).

### The role of phages in regulating the relative abundance of lactation-associated bacteria

**Fig. 7:**
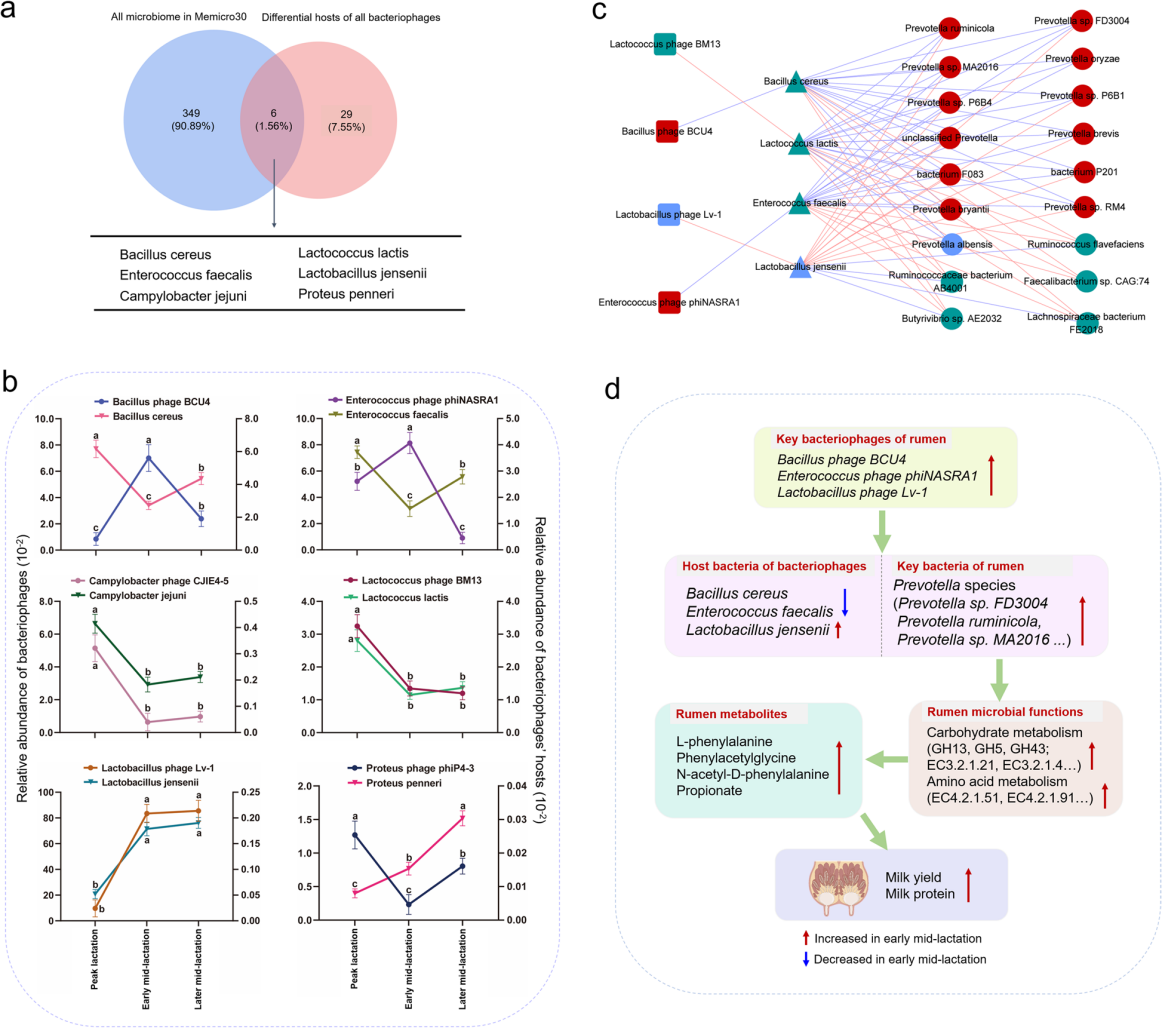
Relationships between rumen phages and their corresponding hosts. **a** Venn diagram of the rumen microbiome in MEmicro 30 and differential hosts of all the bacteriophages. **b** Alterations in differential phages and their differential hosts in MEmicro 30. **c** Spearman correlation network among the phages and their bacterial hosts, as well as differential bacterial species. The colors of nodes are as follows: green, significantly enriched in peak lactation; red, significantly enriched in early mid-lactation; and blue, significantly enriched in later mid-lactation. The shapes of nodes: rectangle, phage; triangle, host matched by the phage; ellipse, microbiome. Line colors: red, positive; blue, negative. Only strong correlations are displayed (|R|> 0.5, *P* < 0.05). **d** Summary diagram of rumen phage and bacterial interactions regulating the milk performance of cows

Generally, considering the rumen phages identified at three lactation stages and specific hosts selected from SEM analysis, a total of 6 critical bacteria were observed among the potential hosts of these phages (Fig. 7a). The associations between the relative abundances of phages and their hosts (Fig. 7b) showed that alteration trends of *Lactococcus phage BM13*, *Campylobacter phage CJIE4-5* (decreasing then stabilizing), and *Lactobacillus phage Lv-1* (increasing then stabilizing) were consistent with those of their corresponding hosts (*Lactococcus lactis*, *Campylobacter jejuni*, and *Lactobacillus jensenii*) (*P* < 0.05). However, the alteration trends of *Bacillus phage BCU4* and *Enterococcus phage phiNASRA1* (increasing then decreasing) were opposite to those of their hosts (*Bacillus cereus* and *Enterococcus faecalis*, decreasing then increasing) (*P* < 0.05).

Next, the relationships among the relative abundances of rumen phages and their matched host, as well as bacterial species, were investigated (Fig. 7c). Results exhibited negative correlations between the abundance of *Enterococcus phage phiNASRA1*, *Bacillus phage BCU4*, and host bacteria (*R* < − 0.50, *P* < 0.05), which were negatively associated with *Prevotella* species but positively correlated with *Ruminococcus flavefaciens*, *Butyrivibrio* sp. *AE2032*, *Ruminococcaceae bacterium AB4001*, *Faecalibacterium* sp. *CAG 74*, and *Lachnospiraceae bacterium FE2018* (|*R*|> 0.50, *P* < 0.05). In contrast, the relative abundance of *Lactococcus phage BM13* and *Lactobacillus phage Lv-1* were positively associated with their host bacteria (*R* > 0.50, *P* < 0.05), and these host bacteria were closely related to *Prevotella* species (|*R*|> 0.50, *P* < 0.05).

## Discussion

In the present study, a large number of bacterial phages were identified in the rumen of dairy cows which had varied shift patterns during the lactation. Previous analyses of the rumen metagenome in steers and goats have demonstrated that diet was a major driver of changes in viral communities [8, 22], and the dairy cattle fed starch- and lipid-rich exhibited higher richness of rumen phages [12]. In this study, although the dietary strategy and management conditions were constant in peak lactation, early, and later mid-lactation, the diversity and relative abundance of the rumen phages detected were distinct, manifesting a trend of first decreasing then increasing or decreasing then stabilizing from peak to later mid-lactation. Notably, we observed that *Enterococcus phage phiNASRA1* and *Bacillus phage BCU4* had the highest abundance in early mid-lactation. *Enterococcus phage phiNASRA1*, belonging to the Siphoviridae family, has been shown to be a highly lytic efficient phage, which is widely present in the ecological environment and gastrointestinal tract [23] and can reduce the number of bacteria by lytic cell activity [24]. However, the biological roles of the *Bacillus phage BCU4* have not been fully elucidated. It has been reported that *Bacillus* sp. are usually allochthonous to the gastrointestinal tract [25] and can be detected in silage [26–28]. Therefore, the *Bacillus cereus* we identified in the rumen may be derived from corn silage in the diet. Since the animals selected in this study were subject to identical feeding management, any observed variation in *Bacillus cereus* can be attributed to changes in the lactation period, although the relative abundance was lower. This may be due to the fact that it is an aerobic or facultative anaerobic bacteria [25]. Most studies conducted to date have focused predominantly on dominant populations in the rumen, with a concomitant neglect of low-abundance microbes. Indeed, despite the low abundance of certain bacteria, such as *Bacillus* species, these keystone taxa play vital roles in shaping the microbial communities of specific niches and improving animal production performance [29–31]. Furthermore, given the capacity of phages to infect and replicate within bacteria, it can be hypothesized that the dynamics of *Bacillus phage BCU4* at distinct phases may contribute to the maintenance of equilibrium within the rumen microbiota by modulating the host bacteria, *Bacillus cereus*. Moreover, we found that the relative abundance of *Lactococcus phage BM13* and *Campylobacter phage CJIE4-5* significantly increased in the peak lactation. *Lactococcus* phages are considered one of the most prevalent phages for infecting bacteria, especially *Lactococcus* species (such as *Lactococcus lactis*), and are capable of modulating the diversity of *Lactococcus* that participate in the body’s energy metabolism [32]. In this study, the increased *Lactococcus phage BM13* and *Lactococcus lactis* in peak lactation may support the characteristics of cows with higher milk yield during this period. Most of studies on *Campylobacter phages* have focused on poultry and food [33–35], and have shown that it can reduce numbers of the foodborne pathogen (mainly *Campylobacter*) in food chain [36, 37]. Their role in the rumen of ruminants is still unclear. Together, these results indicated that a variety of phages were generated in the rumen of dairy cows during the lactation, revealing that rumen phages both are abundant and diverse [20]. Such fluctuations in phage abundance could have significant effects on the stability and functional redundancy of microbial communities. Furthermore, because previous reports of these phages primarily concentrated on humans [24, 32], food [34, 37, 38], and monogastric animals [33, 35], it is necessary to isolate and culture the rumen key phages in the future to better understand their features.

The current study revealed that bacteriome not only showed similar changing patterns (decreasing then increasing or decreasing then stabilizing) to phageome over time, but exhibited first increasing then stabilizing or continuously decline. Similar to our findings, the diversity and community composition of rumen bacteria fluctuated greatly during the lactation period of dairy cows [6, 7, 39], and the richness of rumen bacteria had a decreasing trend from early to late lactation [5]. Interestingly, although there were significant changes in the rumen bacteria of cows at different stages of lactation, these altered bacterial taxa were not exactly the same. For example, the relative abundances of most *Prevotella* species increased in early and later mid-lactation, while *Ruminococcus flavefaciens*, *Ruminococcaceae bacterium AB4001*, *Butyrivibrio* sp. *AE2032*, *Lachnospiraceae bacterium FE2018*, and *Faecalibacterium* sp. *CAG 74* increased in peak lactation. Previous studies have shown that certain bacterial genera, such as *Acetobacter*, *Lactobacillus*, and *Arthrobacter*, were more abundant in mid-lactation compared to late lactation, and they were the main factors leading to variations in bacterial community profiles at different periods [6]. Differences in the identified bacterial taxa may be attributed to the fact that comparisons between different lactation stages. Specifically, we focused on peak (days in milk, DIM = 70 days), early- (DIM = 120 days), and later mid-lactation (DIM = 150 days), while previous research analyzed mid (DIM = 91 d) and late lactation (DIM = 294 days) [6]. Different species within the same genus level may have different functions. Therefore, bacterial taxa differences may also be attributed to the fact that previous assessment of bacterial taxonomy were based only on the genus rather than species level.

The replication and survival of phages depend on the bacterial hosts, and the interactions between them (phages-bacteria) are critical for shaping microbial ecology [19]. We further investigated the relationship between differential rumen phages of dairy cows and their matching bacterial hosts. From peak to later mid-lactation, the relative abundances of *Bacillus phage BCU4* and *Enterococcus phage phiNASRA1* showed opposite trend to those of their host bacteria (*Bacillus cereus* and *Enterococcus faecalis*), implicating that phages predation might affect bacterial communities in a top (phage)–down (bacteria host) manner via cell lysis [13, 15, 40]. Surprisingly, we found that these host bacteria (*Bacillus cereus* and *Enterococcus faecalis*) were negatively correlated with *Prevotella* species. Previous studies have only focused on the matching relationship between phages and their hosts, and showed that the predicted hosts for the most abundant virus families were mainly *Firmicutes*, *Proteobacteria*, and *Bacteroidetes*, while there were few studies comparing the relative abundance of phages with their host bacteria [8, 41]. Recent reports have suggested that rumen microbes with important functions were targeted by viruses (phages), especially *Prevotella* [20–22]. In the present study, although no differential phages infected with *Prevotella* were observed, certain functional phages (*Bacillus phage BCU4* and *Enterococcus phage phiNASRA1*), as previously reported, may promote intense competition (e.g., niche competition or nutrient competition) and cooperation among bacterial species in the gastrointestinal environment [12, 15, 42]. This demonstrated that rumen phage predation not only directly influences target host bacteria, but also produces cascading effects on other bacterial species [15]. In fact, the selective pressure exerted by phages on bacterial communities appears to be a key driver at the population level and beyond that contributes to the diversity and stability of the rumen microbiota via the process of antagonistic coevolution [16, 43]. Moreover, we found that the shift trends of *Campylobacter phage CJIE4-5*, *Lactococcus phage BM13*, and *Lactobacillus phage Lv-1* were consistent with those of their hosts (*Campylobacter jejuni*, *Lactococcus lactis*, and *Lactobacillus jensenii*). Their changes can also consequently be restricted by the presence of prey because phages are specialized parasites of bacterial hosts [14, 19]. Overall, these results suggest that changes of rumen phage abundance from cows at different lactation stages may be involved in the regulation of rumen bacterial abundance alterations, which has rarely been reported before. The phages targeting key bacteria identified can be cultured in vitro with host bacteria in subsequent studies to further verify the potential causality between phages and bacteria by observing changes of host bacteria and other bacterial communities in the culture medium. Although the current research did not focus on archaeaphages [9, 22] or mycophages [44], their interactions with the microbiome might also be a factor influencing ruminal function and lactation performance. This may deserve further investigation in the future.

The present study also revealed that phage predation in rumen microbiota had a potential effect on lactation performance of host, as manifested by the regulation of rumen metabolome. Due to the important role of microbial metabolites in mediating the interaction between bacteria and animal phenotypes [1, 45–47], the regulation of milk composition by rumen microbiome and metabolome was evaluated using SEM. We observed that the microbial module mainly composed of *Prevotella* species had a positive regulatory effect on milk protein by increasing the content of ruminal L-phenylalanine and phenylacetylgcine. Notably, the host bacteria of *Bacillus phage BCU4* and *Enterococcus phage phiNASRA1* were also identified in this microbial module, and the decrease in abundance of *Bacillus cereus* and *Enterococcus faecalis* (host bacteria) favored the increase of *Prevotella* species abundance (Fig. 7c–d). It is reported that *Prevotella* is one of the dominant ruminal genera of dairy cows, and has been demonstrated that they had enzymes and gene clusters are necessary for fermentation and utilization of complex polysaccharides and for facilitating protein breakdown [48, 49]. Our results showed that abundant *Prevotella* species in early mid-lactation were conducive to carbohydrate metabolism to produce propionate and improve milk yield in dairy cows. Moreover, amino acids in the rumen are key precursors for protein and peptide synthesis [50, 51] and are derived from the degradation of feed proteins by rumen microbes. In this study, most *Prevotella* species have a high contribution to amino acid metabolism, and are positively correlated with L-phenylalanine, phenylacetylglycine, and N-acetyl-D-phenylalanine in the rumen, suggesting a possible role of *Prevotella* species in phenylalanine metabolism. Prior research has reported that phenylalanine deficiency had an obvious effect on dairy cow productivity, especially milk protein production [52]. Adding phenylalanine to an amino acid infusion could increase the milk protein concentration [53]. Thus, a higher content of L-phenylalanine in the early mid-lactation may favor the synthesis of milk proteins. These results showed that the increase in relative abundance of key phages during early mid-lactation may be a potential target to rationally regulate microbial metabolism and improve milk protein and yield in dairy cows, which might provide new insights into whether these phages could be manipulated to improve the milk traits of cows.

Phages play an important role in rumen microecology. Although the identification of phages based on metagenomics is effective [54, 55], this sequencing approach extracts the total genome DNA from the sample, including bacteria and archaea, which results in the limited proportion of phages in the generated data [55]. Future studies combining metavirome and metagenome methods may improve phage identification and functional resolution. Additionally, several studies have shown that using phages as antibacterial agents can resist infections caused by pathogens in animals. For instance, phages have been used to control bacterial infections in dairy cows suffering from mastitis [56–58], and it can also reduce contamination of meat and milk [59], thereby improving the quality of dairy products and reducing the occurrence of diseases. Therefore, the key phages identified in this study can be attempted to be applied as supplements in production in the future, with the aim of becoming a possible strategy to regulate rumen microbial metabolism and increase milk production or composition. This is also beneficial for further verifying the causal relationship between phages and bacteria. It is worth noting that the appropriate dosage of phages, supplementation time, and route should be considered. In summary, such information will provide evidence highlighting the possibility that certain phages affect the animal’s lactation performance by manipulating rumen microbial functions and metabolites.

## Conclusion

In summary, rumen phages and bacterial communities exhibited dynamic variations at different lactation stages, and there was a strong correlation between them. Unraveling how key phage-bacteria interactions maintain the stability and function of the rumen microecology during the lactation, phages were associated with changes in their target bacteria, and may have contributed to broader shifts in bacterial communities through cascading associations. In particular, abundant *Bacillus phage BCU4* and the *Enterococcus phage phiNASRA1* in early mid-lactation reduced the relative abundance of their host bacteria, and increased the relative abundance of *Prevotella* species, which enhanced functions related to carbohydrate metabolism and amino acid biosynthesis, contributing to milk yield and protein synthesis. Our study reveals the potential role of the interactions between rumen phages and bacteria in regulating milk properties, and provides new insights into the phageome-microbiome-metabolome-dependent mechanisms of high-quality milk production in dairy cows.

## Methods

### Animal, study design, and sample collection

Sixteen healthy multiparous lactating Holstein cows with comparable body condition scores (body condition scores 2.5–3.0) and lactation stages (60 ± 5 days postpartum) were selected from a cohort of cows housed at a commercial dairy farm in Ning Xia, China (Fig. S1a). Cows were monitored from early to mid-lactation (21–200 days postpartum) and fed a standardized diet with a concentrate-to-forage ratio of 55:45 (dry matter basis). The detailed ingredient compositions and nutrition levels of the feed are presented in Table S5. Animals were milked and fed three times daily (0600, 1400, and 2200 h) with ad libitum access to water. Dry matter intake (DMI) was calculated by recording daily feed consumption and dry matter content. Milk samples were collected for the last 3 consecutive days of every lactation period (68–70, 118–120, and 148–150 DIM, respectively), and the daily milk yield of each cow was recorded through the Afimilk automatic system (Afimilk Ltd., Israel). Then, the average milk yield of these 3 days was respectively used as the representative of each lactation period. Milk samples from individual cows 3 times per day were mixed at a ratio of 4:3:3 (morning, afternoon, and evening), and the milk composition (fat, protein, lactose) of the mixed samples were analyzed using a MilkoScan FT1 instrument (Foss Electric, Denmark).

Rumen fluid samples were collected using oral stomach tubes prior to morning feeding during the peak (70 DIM), early mid- (120 DIM), and later mid-lactation (150 DIM). The initial 100 mL of rumen fluid from each cow was discarded to prevent saliva contamination, and the next 50 mL of rumen fluid was filtered through four layers of sterile cheesecloth in an environment with a steady CO2 flux. Then, each sample was separated into three sections for a subsequent volatile fatty acid (VFA) measurement, microbiome, and metabolome analysis.

### Determination of volatile fatty acids

Volatile fatty acids were determined through the gas chromatograph (Agilent 7820 A, Santa Clara, CA, USA) equipped with a capillary column (AE-FFAP, 30 m × 0.25 mm × 0.33 μm, ATECH Technologies Co., Lanzhou, China) as previously described [60]. In brief, the thawed rumen fluid samples were centrifuged at 13,500 × g for 10 min at 4 °C. The supernatant was mixed with 25% metaphosphoric acid. After standing for 4 h, the mixture was centrifuged at 13,500 × g for 15 min at 4 °C for the separation of protein and impurity. Crotonic acid was added as an internal standard to the supernatant, then filtered using an organic membrane and subsequently transferred it to a gas phase bottle for the detection of VFA.

### DNA extraction, metagenomic sequencing, and data processing

Total genomic DNA was extracted from rumen fluid according to the instructions of the Mag-Bind® Soil DNA Kit (Omega Bio-tek, Norcross, GA, USA). The concentration and quality of DNA were assessed with a NanoDrop2000 spectrophotometer (Thermo Fisher Scientific, MA, USA) and 1% agarose gel electrophoresis.

A total of 18 DNA samples were collected from six out of sixteen selected cows across three distinct lactation periods for metagenomic sequencing. The DNA was fragmented to an average size of approximately 400 bp using a Covaris M220 (Gene Company Limited, China) for the construction of paired-end library. Libraries were prepared with NEXTFLEX® Rapid DNA-Seq Kits (Bioo Scientific, Austin, TX, USA). Adapters containing sequences complementary to the sequencing primer hybridization sites were ligated to the blunt-end of the fragments. Paired-end sequencing was performed on an Illumina NovaSeq platform (Illumina, San Diego, CA, USA) at Majorbio Biopharm Technology Co., Ltd. (Shanghai, China).

Quality control of each dataset was executed using fastp (version 0.20.0, https://github.com/OpenGene/fastp), which involved trimming reads of 3′-end and 5′-end, removing low-quality reads (length < 50 bp or quality scores < 20 or having N bases [61]. Reads were aligned against the bovine genome (bosTau8 3.7, 10.18129/B9.bioc.BSgenome.Btaurus.UCSC.bosTau8) using Burrows-Wheeler Aligner (BWA) (version 0.7.9a, http://biobwa.sourceforge.net) to filter out host DNA [62]. Filtered reads were then de novo assembled for each sample with Megahit (version 1.1.2, https://github.com/voutcn/megahit) [63]. MetaGene (http://metagene.cb.k.u-tokyo.ac.jp/) was utilized to predict open reading frames (ORFs) from assembled contigs longer than 300 bp [64]. The ORFs with a length ≥ 100 bp were retrieved and translated into amino acid sequences. The assembled contigs were then pooled and non-redundant gene catalog was generated applying CD-HIT (version 4.6.1, http://www.bioinformatics.org/cd-hit/) with 90% sequence identity and 90% coverage [65]. High-quality reads were aligned to the non-redundant gene catalogs to calculate gene abundance with 95% identity using SOAPaligner (version 2.21, http://soap.genomics.org.cn/) [66].

Taxonomic assessment of ruminal bacteria and archaea was performed against the nonredundant (NR) database using the blastp alternative in Diamond (version 0.8.35, http://www.diamondsearch.org/index.php), with an e-value of 1e^−5^ [67]. Alpha diversity of rumen microbial communities was calculated based on Mothur software (version 1.30.2, https://mothur.org/wiki/calculators/). Principal coordinate analysis (PCoA) was conducted based on Bray–Curtis dissimilarity matrices at the species level. Shift patterns in phage and bacterial profiles during different stages of lactation were analyzed by the Short Time-series Expression Miner (STEM, version 1.3.11, http://www.cs.cmu.edu/~jernst/stem) [68]. Rumen phage dynamics over time were visualized using the R package BiomeHorizon (version 1.0.0, https://github.com/blekhmanlab/biomehorizon/) [69]. Identification of rumen bacteriophages and prediction of phage-host relationship were achieved using Diamond (version 0.8.35) based on the GenomeNet Virus-Host Database (http://www.genome.jp/virushostdb/), with an e-value threshold setting 1e^−5^ for the blastn alignment [67, 70].

The functional annotation was performed by Diamond (version 0.8.35) according to the Kyoto Encyclopedia of Genes and Genomes (KEGG, http://www.genome.jp/kegg/) databases (using blastp with an e-value of 1e^−5^) [67]. Carbohydrate-active enzyme (CAZymes) annotation was performed using hmmscan (version 3.1b2, http://hmmer.janelia.org/search/hmmscan) against the CAZyes database (http://www.cazy.org/), with an e-value of 1e^−5^. The relative abundances of KEGG pathways, gene enzymes, and CAZymes were normalized into reads per kilobase million (RPKM) for downstream analysis.

### Metabolomic analysis

Metabolomic analysis was conducted on rumen fluid samples collected from six cows across three distinct lactation stages. All sample scans were performed using a liquid chromatography-mass spectrometry system, adhering to the manufacturer’s guidelines. Briefly, approximately 100 μL of rumen fluid was transferred into a 1.5-mL centrifuge tube containing 400 μL of a solution composed of acetonitrile and methanol (1:1, v/v), supplemented with 0.02 mg/mL of the internal standard L-2-chlorophenylalanine. The samples were mixed by vortex for 30 s and ultrasonication at 40 kHz for 30 min at 5 °C, followed by placing them at − 20 °C for 30 min to precipitate proteins. The samples were centrifuged at 13,000 × g for 15 min at 4 °C. After being taken out, the supernatant was blown dry using nitrogen. The concentrated product was re-solubilized in 100 μL of water/acetonitrile (1:1, v/v), and extracted in an ultrasonic bath at 5 °C with the ultrasonic frequency of 40 kHz for 5 min. After centrifuging the mixture, the supernatant was then carefully transferred to sample vials for LC‒MS/MS analysis. Additionally, as part of the system conditioning and quality control process, a pooled quality control (QC) sample was prepared by mixing equal volumes of all samples and treated identically to the experimental samples.

Chromatographic separation of metabolites was carried out on a Thermo UHPLC-Q Exactive system equipped with an ACQUITY HSS T3 column (100 × 2.1 mm i.d., 1.8 µm; Waters, Milford, USA). The mobile phases consisted of solvent A (0.1% formic acid in water:acetonitrile, 95:5, v/v) and solvent B (0.1% formic acid in acetonitrile:isopropanol:water, 47.5:47.5:5, v/v/v). The injection volume was 2.0 µL, with a flow rate of 0.4 mL/min, and the column temperature was maintained at 40 °C. During the analysis period, all samples were stored at 4 °C. The metabolomics processing software Progenesis QI (Waters Corporation, Milford, USA) was utilized to analyze the LC–MS raw data. Only variables with more than 80% nonzero values in at least one group were retained from the extracted ion features. Metabolite identification was achieved by mapping to the HMDB metabolic public database (http://www.hmdb.ca/) and Metlin (https://metlin.scripps.edu/).

Following normalization to total peak intensity, supervised partial least squares discriminant analysis (PLS-DA) was conducted using the R package ropls (version 1.6.2) to distinguish the different variables among the stages of lactation. Differential metabolites were summarized and mapped to their biochemical pathways through metabolic enrichment and pathway analysis based on KEGG database (http://www.genome.jp/kegg/). The Python package SciPy (version 1.0.0) was used for pathways enrichment analysis, and the statistical significance of biological pathways was identified via Fisher’s exact test.

### Weighted gene coexpression network analysis

WGCNA was employed to identify key phenotype-related metagenomic and metabolic modules based on their correlation patterns. The analysis was conducted using the R packages WGCNA (version 1.68) [71] and vegan (version 2.5.6) [72], following guidelines from official tutorials (https://horvath.genetics.ucla.edu). To fully describe the microbiota-metabolite-milk trait metabolic network features, peripheral and central metabolites were integrated into a scale-free network topology, with abundance data normalized through logarithmic conversion and robust quantile normalization. A “step-by-step network construction” method was applied for the metabolic network topology. The network type was set to “signed hybrid,” with soft thresholding powers of 6 for the rumen microbiome (Fig. S9a) and 14 for the rumen metabolome (Fig. S9b), optimizing the topological overlap matrix. Other parameters were kept at default settings. Genes were clustered using the average linkage hierarchical clustering method in Hclust, and expression modules were detected using dynamicTreeCut. Modules with similar patterns were further clustered and merged into consensus modules. The correlation between these consensus modules and milk composition was calculated using corPvalueStudent. Pairwise Pearson correlation coefficients were computed for all selected microbes/metabolites, converting the resulting Pearson correlation matrix into a matrix of connection strengths (an adjacency matrix) via a power function. This adjacency matrix was then transformed into a topological overlap matrix. WGCNA aims to identify densely interconnected microbe/metabolite modules through hierarchical clustering based on topological overlap. Metabolites within each module were annotated using the Human Metabolome Database (http://www.hmdb.ca/) and the KEGG database (http://www.genome.jp/kegg/). Annotated metabolites were summarized and mapped to their biochemical pathways through metabolic enrichment and pathway analysis based on the KEGG database. This comprehensive approach facilitates a deeper understanding of the relationships between microbial communities, metabolites, and milk composition.

### Structural equation modeling construction analysis

To assess the direct associations among the rumen microbiome, rumen metabolome, and milk composition, we employed the lavaan package (version 0.5–12) in R to construct a structural equation model (SEM) [73]. Specifically, WGCNA was utilized to modularize the rumen metagenomic and metabolomic data, enabling us to identify key modules that influence milk composition based on relationships established through SEM. The fit of the SEM was evaluated using the *χ*^2^ test, root mean square error of approximation (RMSEA), and comparative fit index (CFI). A model was deemed well fitted if the CFI approached 1 and the statistical *P* values were above the conventional threshold of 0.05 [74]. The SEM construction was guided by the fit indices between different omics modules. When the SEM demonstrated robust fit indices, it enabled the interpretation of path coefficients and their corresponding *P* values. Path coefficients, akin to partial correlation coefficients, indicate both the magnitude and direction of relationships between variables. These coefficients provided insights into how variations in the rumen microbiome and metabolome impact milk composition. By analyzing these path coefficients, we could uncover the underlying mechanisms driving these relationships within the context of the model’s structure. This approach facilitated a deeper understanding of the intricate interactions among the rumen microbiome, metabolome, and milk composition.

### Statistical analysis

The differences in lactation performance and rumen fermentation parameters were compared using one-way analysis of variance (ANOVA). The Kruskal–Wallis *H* test with Dunn’s post hoc test was employed to analyze distinctions in microbial alpha diversity among the different stages of lactation. ANOSIM based on Bray–Curtis distance matrices was used to determine the beta diversity between two and more compared groups. The distinctions of microbial species were conducted by the Kruskal–Wallis *H* test, with post hoc analysis performed using Tukey–Kramer. Linear discriminant analysis effect size (LEfSe) was performed to compare the relative abundance of microbial metabolic pathways and CAZymes, and significant differences were defined by a LDA > 2 and *P* < 0.05. Relationships among the rumen microbes, gene enzymes, rumen metabolites, and milk compositions were explored using Spearman’s rank correlation test, and |R|> 0.5, *P* < 0.05, was used to identify significant correlations. The visualization of the network structure was performed by Cytoscape 3.7.2.

## Supplementary Information

Supplementary Material 1: Fig. S1 Schematic diagram of the experimental design and the rumen microbial diversity of dairy cows at different lactation stages. a Experimental design. The red arrow represents the sampling time point. Ace (b) and Shannon (c) indices of rumen archaea at the species level. d Rumen archaeal signatures at different lactation stages based on species visualized using principal coordinate analysis (PCoA). *P* values were determined using the nonparametric Kruskal-Wallis test. *P < 0.05.

Supplementary Material 2: Fig. S2 Rumen microbial composition profiles of dairy cows at different lactation stages. Rumen phage (a), bacterial (c), and archaeal (e) compositions at the species level. The shift patterns of rumen phages (b) and bacteria (d) as changes of the lactation stage. In each frame, the profile ID is presented on the top left, the number of phages or bacteria is shown on the bottom left, the x-axis indicates the stages of lactation, and the y-axis denotes the abundance of phages or bacteria. The colored frame shows that the number of allocated phages or bacteria with statistical significance, *P* < 0.05. The same color indicates similar transfer patterns of phages or bacteria at different lactation stages. Significantly different archaea (f) at different lactation stages. * indicates a difference at *P* < 0.05.

Supplementary Material 3: Fig. S3 Principal coordinate analysis (PCoA) of rumen microbial function. a Visualization of the KEGG pathways at different lactation stages. b Visualization of the carbohydrate-active enzymes (CAZymes) composition based on family-level at different lactation stages.

Supplementary Material 4: Fig. S4 Differential carbohydrate-active enzymes (CAZymes) in the rumen of dairy cows at different lactation stages. Significant differences were tested by linear discriminant analysis effect size (LEfSe) analysis, with a linear discriminant analysis (LDA) score > 2 and a *P* value < 0.05.

Supplementary Material 5: Fig. S5 Alterations in the rumen metabolomic profiles of dairy cows at different lactation stages. a Partial least squares discriminant analysis (PLS-DA) of the rumen metabolome. b Clustered heatmap of the relative abundances of differential rumen metabolites (top50). The color indicates the relative abundance of the metabolite during the lactation stages. The corresponding relationship between the color gradient and the value is shown in the gradient color block. The samples are shown in columns, and the metabolites are shown in rows.

Supplementary Material 6: Fig. S6 Comparison of phenotypic data of dairy cows at different lactation stages. Dry matter intake (DMI).

Supplementary Material 7: Fig. S7 a Cluster dendrogram of rumen metagenome based on species. b Cluster dendrogram of the rumen metabolome.

Supplementary Material 8: Fig. S8 Enrichment pathways of different metabolic modules in the rumen of dairy cows. a KEGG pathway enrichment analysis of ruminal metabolome module 1 (ruMetab 1). b KEGG pathway enrichment analysis of ruminal metabolome module 3 (ruMetab 3).

Supplementary Material 9: Fig. S9 Soft-thresholding power to 6 (rumen microbiome) based on the species level (a) and (b) 14 (rumen metabolome).

Supplementary Material 10: Table S1. Summary of sequence data generated from rumen fluid samples of dairy cows at different lactation stages.

Supplementary Material 11: Table S2. Rumen fermentation parameters of dairy cows at different lactation stages.

Supplementary Material 12: Table S3. The metabolites of rumen metabolome module 1 based on WGCNA.

Supplementary Material 13: Table S4. The metabolites of rumen metabolome module 3 based on WGCNA.

Supplementary Material 14: Table S5. Ingredients and nutrient levels of total mixed ration.

## Data Availability

The sequencing reads of metagenome are available in the Sequence Read Archive (SRA) of NCBI under accession numbers PRJNA885216. The raw data of metabolome is deposited into the MetaboLights repository (https://www.ebi.ac.uk/metabolights/MTBLS12816).
