# Supplementary figures and images for: Unraveling the interaction between the phageome and bacteriome in the rumen and its role in influencing metabolome dynamics in dairy cows at different lactation stages
Source: Microbiome. 2025 Dec 15;13:257. doi: 10.1186/s40168-025-02260-1 (PMC12739858; doi:10.1186/s40168-025-02260-1)

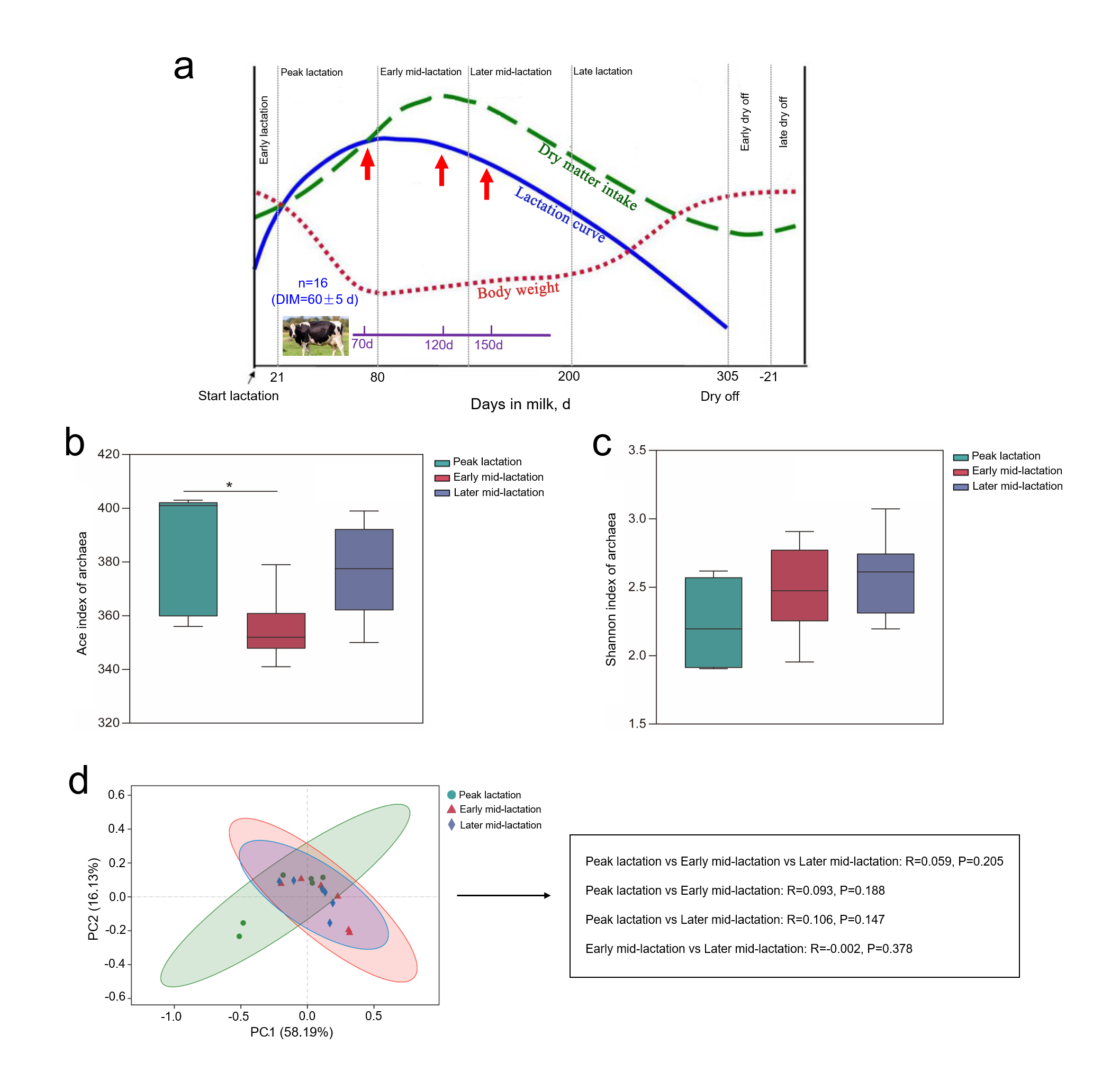

Supplement: Supplementary file 2 — Supplementary Material 1: Fig. S1 Schematic diagram of the experimental design and the rumen microbial diversity of dairy cows at different lactation stages. a Experimental design. The red arrow represents the sampling time point. Ace (b) and Shannon (c) indices of rumen archaea at the species level. d Rumen archaeal signatures at different lactation stages based on species visualized using principal coordinate analysis (PCoA). P values were determined using the nonparametric Kruskal-Wallis test. *P < 0.05. [file 40168_2025_2260_MOESM1_ESM.tif]

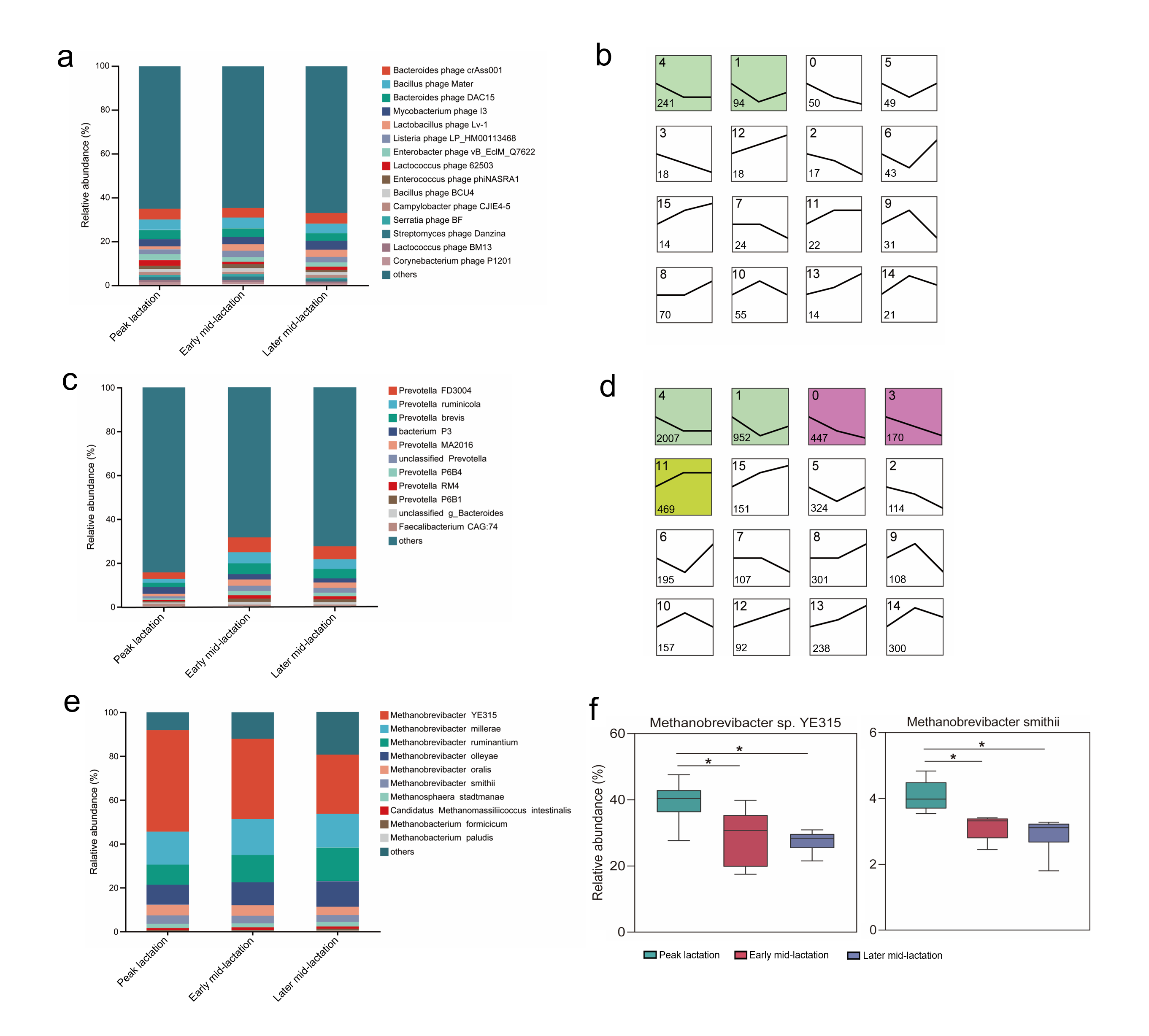

Supplement: Supplementary file 3 — Supplementary Material 2: Fig. S2 Rumen microbial composition profiles of dairy cows at different lactation stages. Rumen phage (a), bacterial (c), and archaeal (e) compositions at the species level. The shift patterns of rumen phages (b) and bacteria (d) as changes of the lactation stage. In each frame, the profile ID is presented on the top left, the number of phages or bacteria is shown on the bottom left, the x-axis indicates the stages of lactation, and the y-axis denotes the abundance of phages or bacteria. The colored frame shows that the number of allocated phages or bacteria with statistical significance, P < 0.05. The same color indicates similar transfer patterns of phages or bacteria at different lactation stages. Significantly different archaea (f) at different lactation stages. * indicates a difference at P < 0.05. [file 40168_2025_2260_MOESM2_ESM.tif]

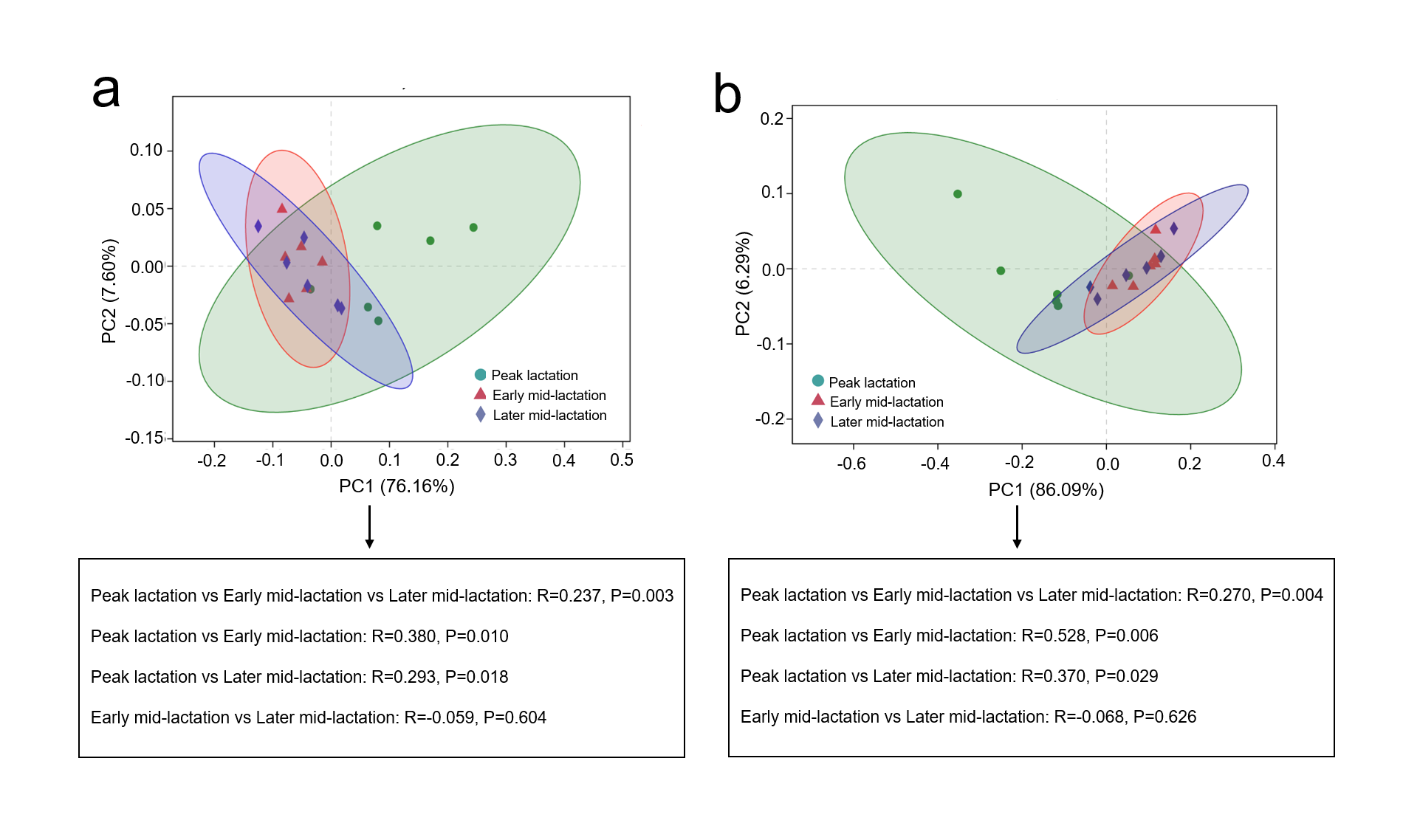

Supplement: Supplementary file 4 — Supplementary Material 3: Fig. S3 Principal coordinate analysis (PCoA) of rumen microbial function. a Visualization of the KEGG pathways at different lactation stages. b Visualization of the carbohydrate-active enzymes (CAZymes) composition based on family-level at different lactation stages. [file 40168_2025_2260_MOESM3_ESM.tif]

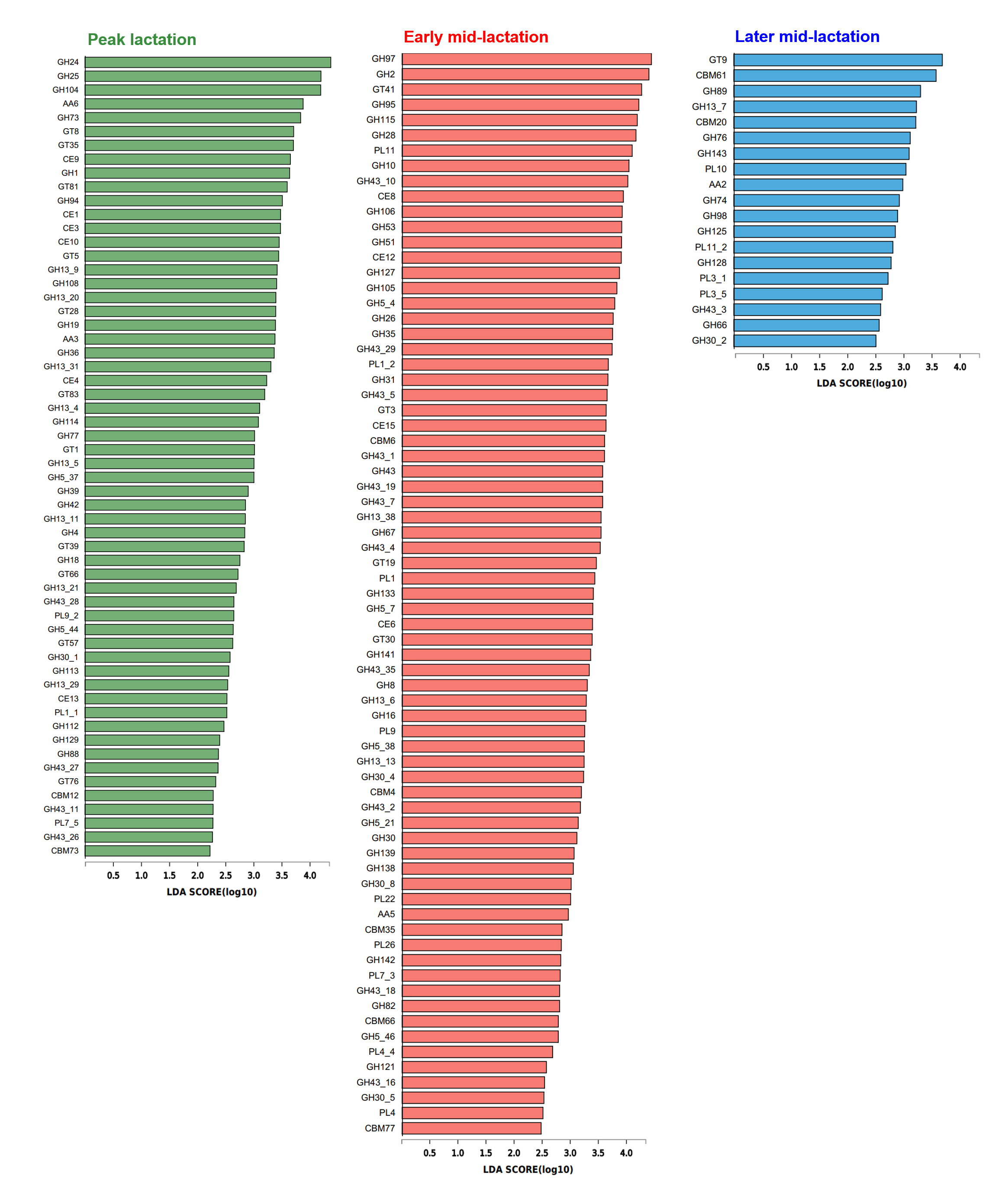

Supplement: Supplementary file 5 — Supplementary Material 4: Fig. S4 Differential carbohydrate-active enzymes (CAZymes) in the rumen of dairy cows at different lactation stages. Significant differences were tested by linear discriminant analysis effect size (LEfSe) analysis, with a linear discriminant analysis (LDA) score > 2 and a P value < 0.05. [file 40168_2025_2260_MOESM4_ESM.tif]

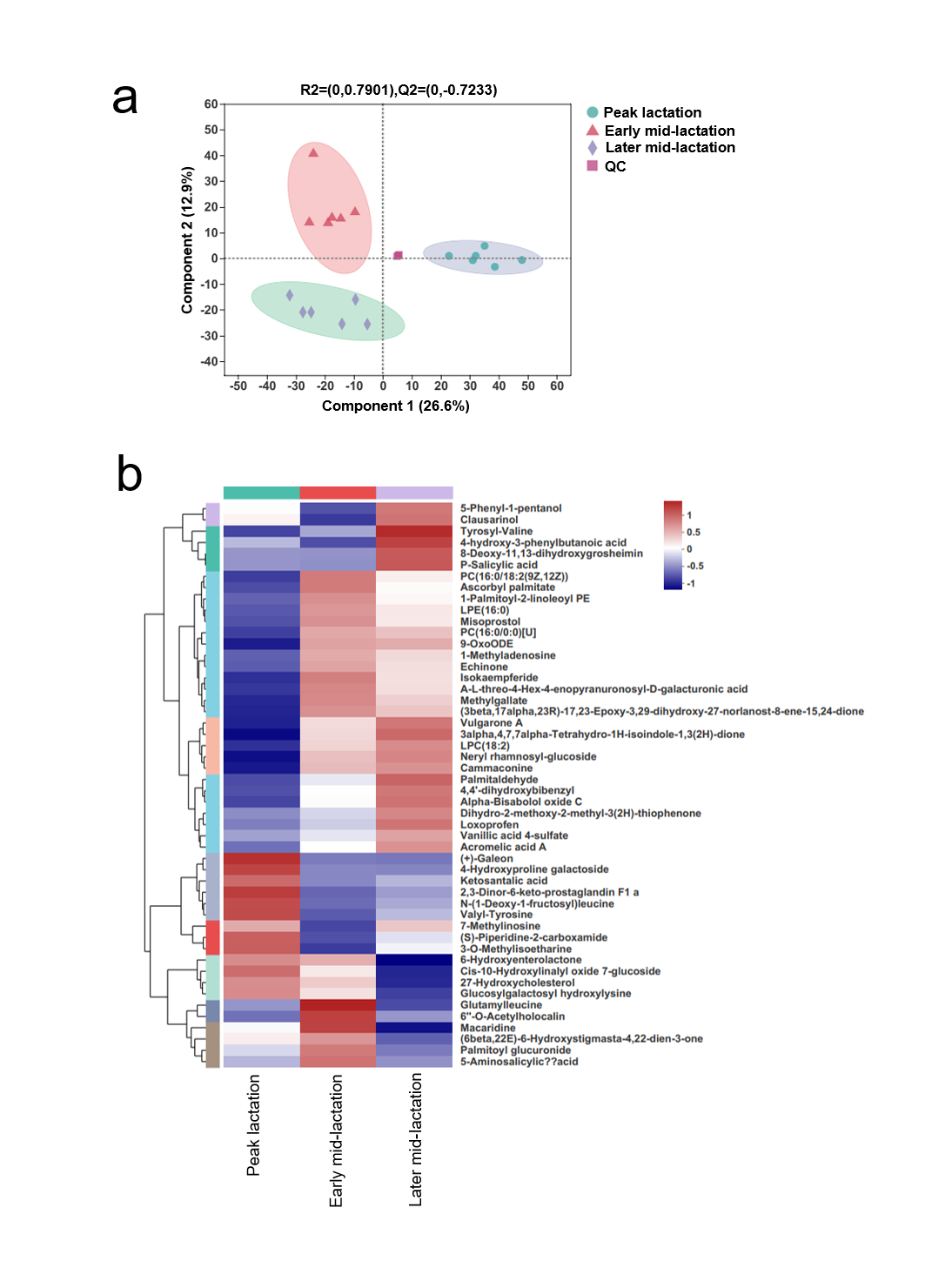

Supplement: Supplementary file 6 — Supplementary Material 5: Fig. S5 Alterations in the rumen metabolomic profiles of dairy cows at different lactation stages. a Partial least squares discriminant analysis (PLS-DA) of the rumen metabolome. b Clustered heatmap of the relative abundances of differential rumen metabolites (top50). The color indicates the relative abundance of the metabolite during the lactation stages. The corresponding relationship between the color gradient and the value is shown in the gradient color block. The samples are shown in columns, and the metabolites are shown in rows. [file 40168_2025_2260_MOESM5_ESM.tif]

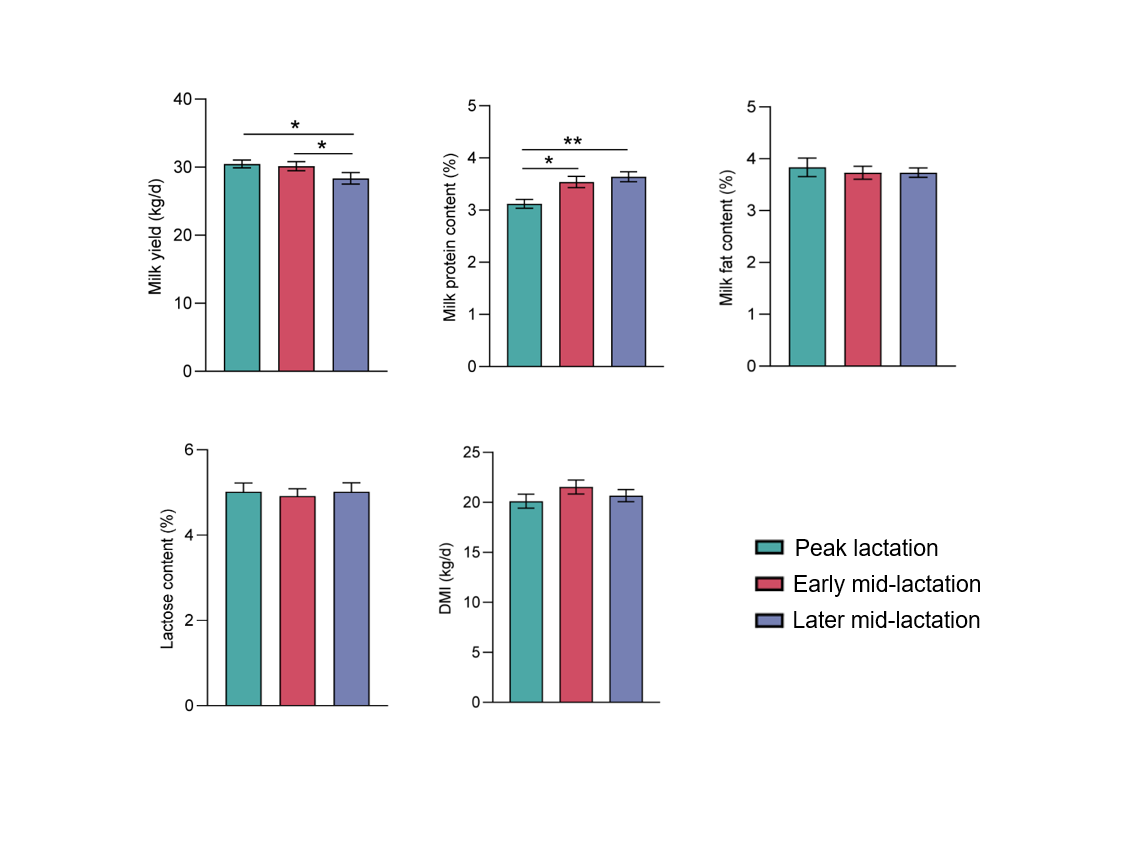

Supplement: Supplementary file 7 — Supplementary Material 6: Fig. S6 Comparison of phenotypic data of dairy cows at different lactation stages. Dry matter intake (DMI). [file 40168_2025_2260_MOESM6_ESM.tif]

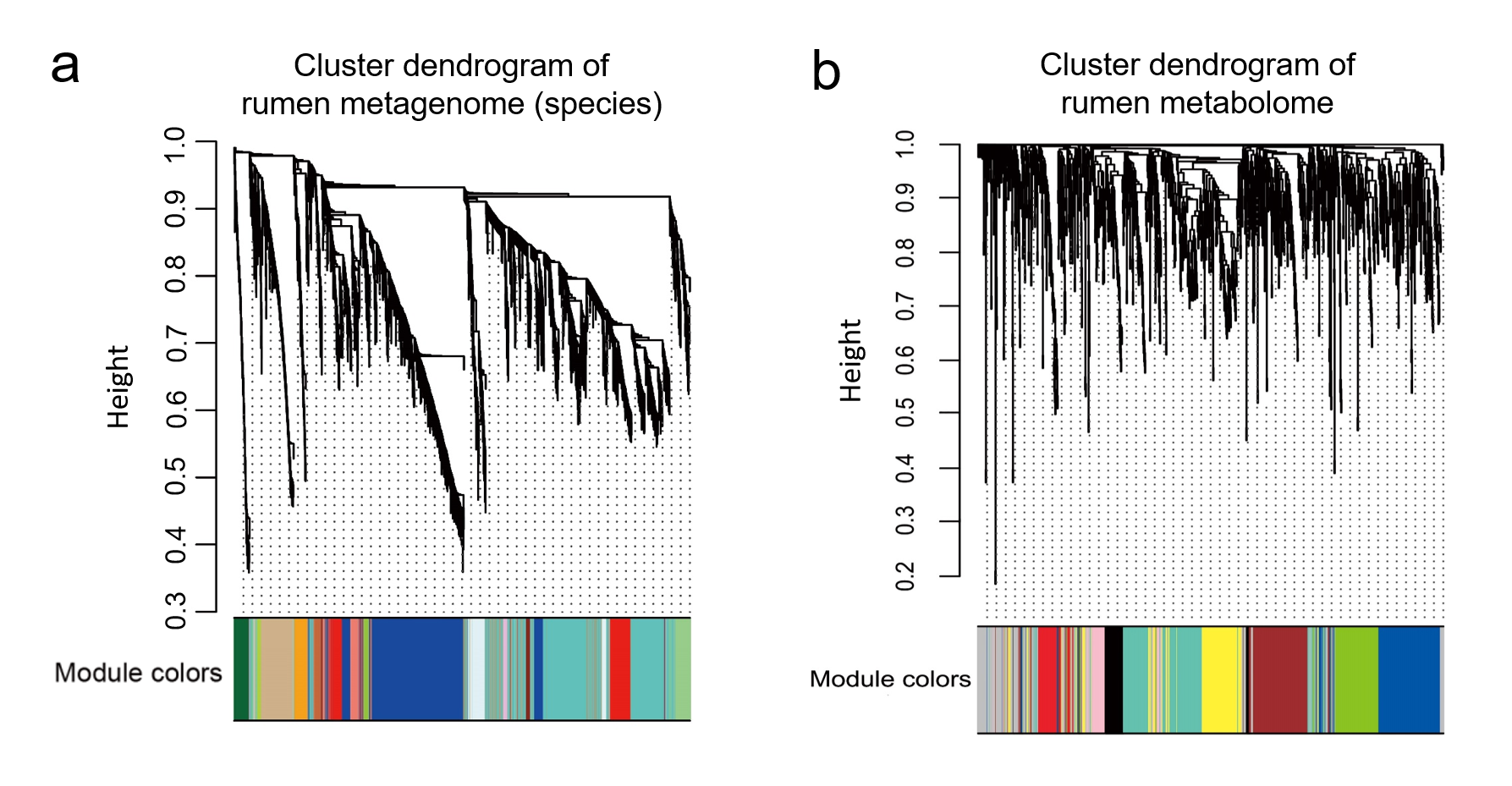

Supplement: Supplementary file 8 — Supplementary Material 7: Fig. S7 a Cluster dendrogram of rumen metagenome based on species. b Cluster dendrogram of the rumen metabolome. [file 40168_2025_2260_MOESM7_ESM.tif]

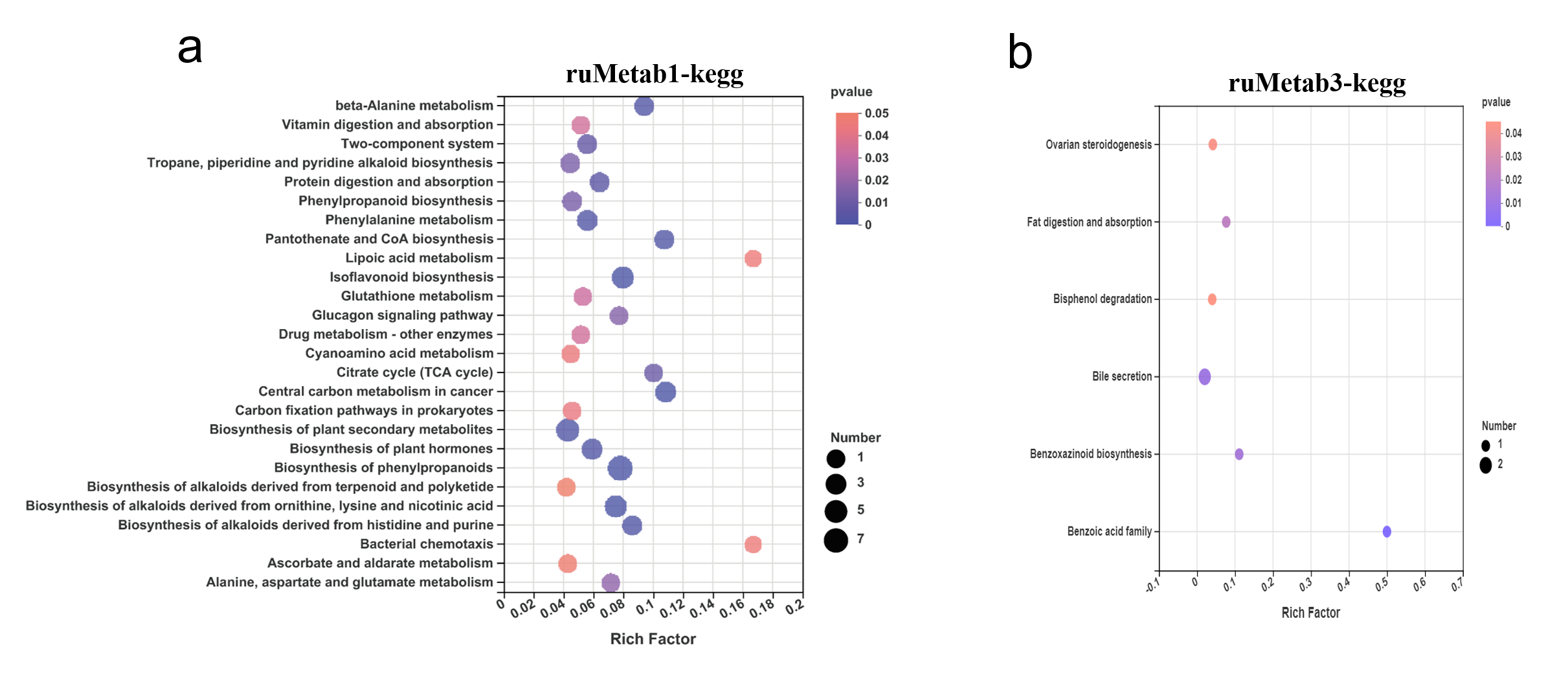

Supplement: Supplementary file 9 — Supplementary Material 8: Fig. S8 Enrichment pathways of different metabolic modules in the rumen of dairy cows. a KEGG pathway enrichment analysis of ruminal metabolome module 1 (ruMetab 1). b KEGG pathway enrichment analysis of ruminal metabolome module 3 (ruMetab 3). [file 40168_2025_2260_MOESM8_ESM.tif]

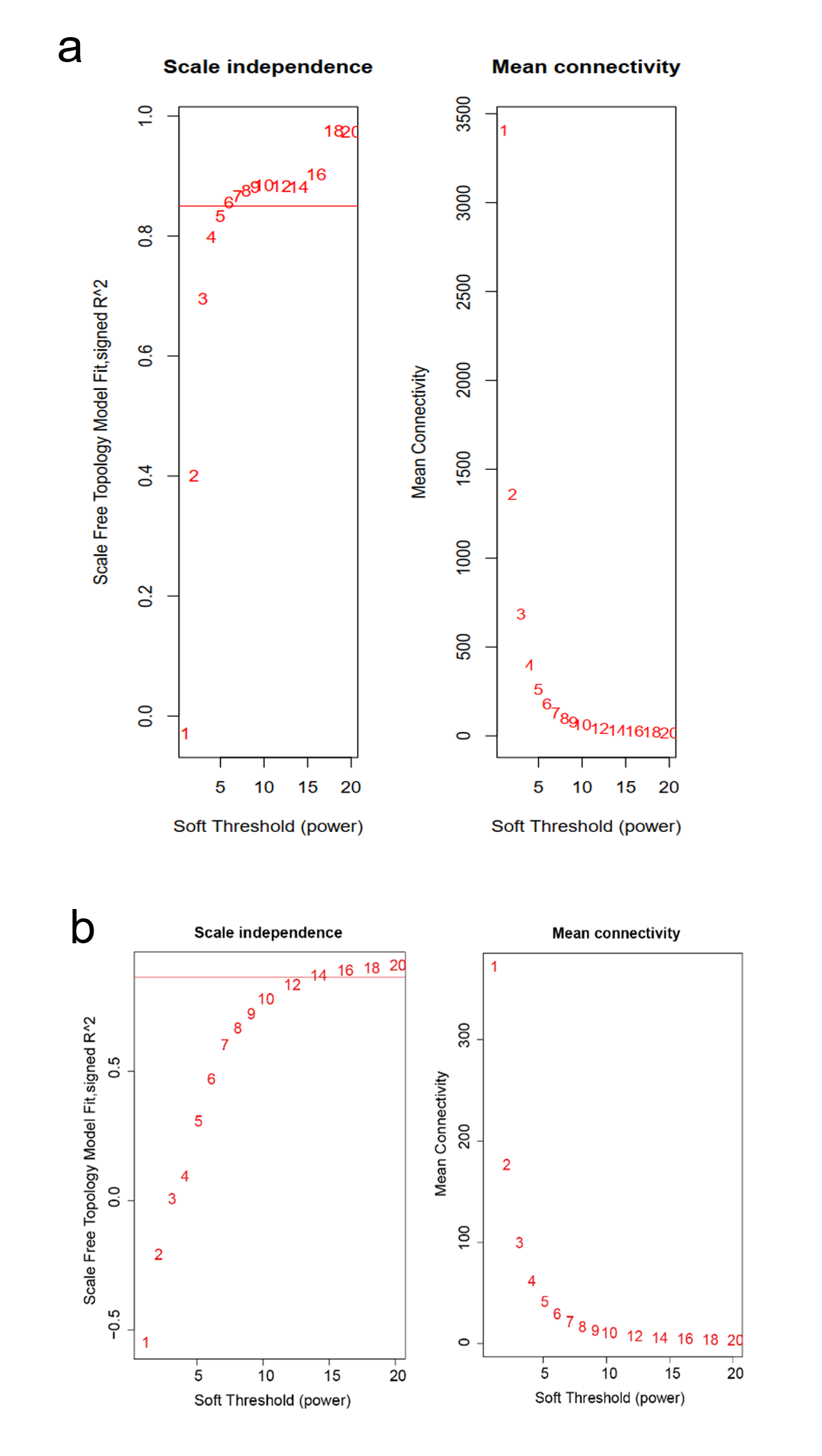

Supplement: Supplementary file 10 — Supplementary Material 9: Fig. S9 Soft-thresholding power to 6 (rumen microbiome) based on the species level (a) and (b) 14 (rumen metabolome). [file 40168_2025_2260_MOESM9_ESM.tif]
