# Supplementary material for: Unraveling the interaction between the phageome and bacteriome in the rumen and its role in influencing metabolome dynamics in dairy cows at different lactation stages
Source: Microbiome. 2025 Dec 15;13:257. doi: 10.1186/s40168-025-02260-1 (PMC12739858; doi:10.1186/s40168-025-02260-1)
Supplement: Supplementary file 12 — Supplementary Material 11: Table S2. Rumen fermentation parameters of dairy cows at different lactation stages. [file 40168_2025_2260_MOESM11_ESM.docx]

| Table S2. Rumen fermentation parameters of dairy cows at different lactation stages. | | | | | |
| --- | --- | --- | --- | --- | --- |
| VFA proportion  (mol/100 mol) | Peak lactation | Early mid-lactation | Later  mid-lactation | SEM | P-value |
| Acetate | 61.32^a^ | 58.03^b^ | 59.75^b^ | 0.630 | 0.018 |
| Propionate | 24.09^ab^ | 26.95^a^ | 23.58^b^ | 0.689 | 0.030 |
| Butyrate | 11.36 | 11.38 | 12.59 | 0.401 | 0.203 |
| Isobutyrate | 0.67^b^ | 0.82^a^ | 0.96^a^ | 0.052 | 0.034 |
| Valerate | 1.33^b^ | 1.46^ab^ | 1.67^a^ | 0.063 | 0.045 |
| Isovalerate | 1.21 | 1.35 | 1.40 | 0.089 | 0.237 |

SEM, stand error of the mean.

*P*-values were calculated using ANOVA (n=6 per ruminant species).

Different superscripts in a row designate significant difference (*P* < 0.05).
