# Supplementary material for: Unraveling the interaction between the phageome and bacteriome in the rumen and its role in influencing metabolome dynamics in dairy cows at different lactation stages
Source: Microbiome. 2025 Dec 15;13:257. doi: 10.1186/s40168-025-02260-1 (PMC12739858; doi:10.1186/s40168-025-02260-1)
Supplement: Supplementary file 15 — Supplementary Material 14: Table S5. Ingredients and nutrient levels of total mixed ration. [file 40168_2025_2260_MOESM14_ESM.docx]

| Table S5. Ingredients and nutrient levels of total mixed ration | | | |
| --- | --- | --- | --- |
| Ingredients,  % of dry matter (DM) | Content | Nutrition levels^2^,  (% of DM, unless other stated) | Content |
| Alfalfa hay | 11.94 | DM | 49.10 |
| Corn silage | 33.06 | Net energy for lactation, NE_L_ (Mcal/kg) | 1.75 |
| Steam-flaked corn | 7.23 | Crude protein, CP | 16.50 |
| Corn flour | 10.85 | Starch | 25.00 |
| Corn gluten powder | 0.93 | Neutral detergent fiber, NDF | 31.00 |
| Corn husk | 4.10 | Acid detergent fiber, ADF | 18.80 |
| Beet pulp | 5.79 | Rumen degradable protein, RDP | 67.20 |
| Soybean meal | 10.85 | Rumen undegradable protein, RUP | 32.80 |
| Cottonseed meal | 1.83 |  |  |
| Whole cottonseed | 5.43 |  |  |
| Fatty powder | 1.01 |  |  |
| NaHCO3 | 1.12 |  |  |
| Detoxin | 0.04 |  |  |
| Yeast | 3.62 |  |  |
| Premix^1^ | 2.19 |  |  |
| ^1^Premix contain (per kilogram): VA 400 KIU, VD_3_ 80 KIU, VE 2200 IU, Fe 1500 mg, Cu 700 mg, Mn 3000 mg, Zn 3200 mg, Se 20 mg, I 70 mg, Co 40 mg, Ca 110 g, P 4 g。  ^2^NE_L,_ RDP, and RUP are calculated values based on CPM software, the rest are measured values. | | | |
